# Protocol for the process evaluation of a mobile produce market intervention to increase fruit and vegetable consumption in lower-income communities: the Veggie Van Study

**DOI:** 10.3389/fpubh.2026.1760383

**Published:** 2026-04-15

**Authors:** Christina Kasprzak, Andy Canizares, Anne Lally, Leah N. Vermont, Laurene Tumiel-Berhalter, Samina Raja, Lindsey Haynes-Maslow, Alice Ammerman, Lucia A. Leone

**Affiliations:** 1 Department of Community Health and Health Behavior, University at Buffalo, Buffalo, NY, United States; 2 Department of Anthropology, University at Buffalo, Buffalo, NC, United States; 3 Department of Family Medicine, University at Buffalo, Buffalo, NC, United States; 4 Department of Urban and Regional Planning, University at Buffalo, Buffalo, NC, United States; 5 Department of Health Policy and Management, The University of North Carolina at Chapel Hill, Chapel Hill, NC, United States; 6 Department of Nutrition, The University of North Carolina at Chapel Hill, Chapel, Hill, NC, United States

**Keywords:** community-based intervention, implementation evaluation, lower-income, mobile market, process evaluation

## Abstract

**Introduction:**

Mobile produce markets have become increasingly prevalent throughout the United States, highlighting the need for evidence-based models for markets to follow. The Veggie Van (VV) model is a set of practices that have been found to be efficacious in increasing fruit and vegetable intake in underserved communities. The next step in determining the model’s effectiveness is evaluating the model when implemented on a broader scale under real-world conditions. As part of a multi-state randomized controlled effectiveness trial, the Veggie Van Study, an extensive process evaluation of the implementation of the VV model was conducted.

**Methods:**

Nine partner organizations agreed to implement the VV model at newly launched mobile markets over a 12-month period. Organizations received funding, training, and technical assistance to support implementation of the model. In addition to facilitating participant recruitment and data collection, partners agreed to participate in implementation-related data collection. Data collection methods include quantitative process measures surveys, qualitative interviews, and mobile market sales data. The main process outcomes are fidelity, dose delivered, penetration, maintenance of the VV model, and contextual factors related to implementation.

**Discussion:**

This extensive mixed-methods process evaluation addresses a gap in the literature for transparent reporting of process evaluations; it also provides a model for similar community-based interventions to follow. Understanding the implementation process and the context surrounding implementation of the VV model is critical for interpreting effectiveness findings (i.e., dietary changes) and optimizing the intervention.

**Clinical trial registration:**

https://clinicaltrials.gov/study/NCT04246593, NCT04246593.

## 1 Introduction

Public health interventions have increasingly prioritized underserved communities to help narrow disparities in diet-related chronic disease. Notably, fruit and vegetable (F&V) consumption is significantly lower in these communities which contributes to higher chronic disease risk (1). While most adults in the United States fail to meet recommended daily intake for F&V, lower-income and/or some minority households have even lower consumption (2, 3). Limited healthy food access is a recognized contributor to low F&V consumption (1, 4); however, not all food access strategies have consistently demonstrated effectiveness in improving F&V consumption (5). Mobile produce markets, or mobile markets, are a popular strategy to address low food access; mobile markets travel to predominantly lower-income or low food access communities to sell affordable foods (6–8). Randomized controlled trials of mobile markets have shown an increase in F&V intake ranging from ½ to 1 cup per day over a period of 6–12 months (9, 10). Compared to other common food access programs, such as community gardens and healthy corner store programs, mobile markets are a favored program among lower-income communities if they are conveniently located and produce is competitively priced (7, 8, 11).

### 1.1 The Veggie Van model

The increasing prevalence of mobile markets across the country is encouraging for improving food access; however, mobile market models vary, and organizations face numerous challenges to operate and sustain them (12). Adopting evidence-based practices can improve operations, facilitate longevity, and increase the likelihood of a mobile market positively impacting diet. The Veggie Van (VV) model is an evidence-based model intended for mobile markets that has been found efficacious in increasing F&V consumption (10). The model is informed by the Social Cognitive Theory which posits the concept of reciprocal determinism to describe the interdependent influences among individuals, their behaviors and the environments in which they live (13). This theory suggests that changing the food environment alone is not enough, but that we also need to address how individuals perceive and interact with that environment. The VV model addresses both the food environment as well as the individual to influence F&V consumption. In addition, the VV model was designed to address multiple dimensions of access to healthy food acceptability, availability, affordability, accessibility, and accommodation (14). The model advises mobile market practitioners to (1) operate the mobile market regularly at convenient locations selected based on their partnerships with community organizations reaching the target population, (2) procure a variety of fresh, high quality F&V, prioritizing local produce as much as possible, (3) adopt a reduced cost payment model selling produce at competitive prices and increase affordability through participation in incentive programs (e.g., Supplemental Nutrition Assistance Program, produce prescription, SNAP matching; etc.), (4) encourage and incentivize customers to purchase a bundle of produce (multiple items for a set price), and (5) offer regular food and nutrition education (e.g., cooking demonstrations, nutrition lessons).

Implementation science underscores the importance of implementing an intervention with fidelity, or how it was intended to be implemented, to ensure that the intervention remains effective in real-world settings (15). Many mobile markets face challenges in routine practice such as issues surrounding financial sustainability and organizational capacity (12, 16, 17). These persistent challenges may undermine an organization’s capacity to implement an evidence-based intervention, such as the VV model. To address these implementation challenges, this research seeks to understand fidelity to the VV model among implementing organizations, factors that help or hinder implementation, and adaptations that preserve the fidelity of the model while suiting the needs of organizations and the communities they serve. Conducting an extensive process evaluation is especially important for this multistate trial in which the VV model is likely implemented in vastly different ways (18). By furthering our understanding of implementation outcomes and factors, we can refine the intervention itself and improve our implementation tools. In addition, we can provide the necessary context for interpreting the results of our main dietary outcomes which will be reported separately.

### 1.2 Veggie Van Study overview

The VV model was recently evaluated in a 7-year hybrid type 1 effectiveness implementation study, known as the Veggie Van (VV) study. Building on the work of the past efficacy study (10), this cluster randomized controlled trial (RCT) evaluated whether the VV model is effective on a broader scale when implemented by multiple community partners across five states in the Eastern US (19). Nine partner organizations were identified through a request-for-partners (RFP) process and received funding to start or expand mobile market operations following the VV model. Details regarding recruitment of partners through the RFP process are reported elsewhere (20). Each of the nine partner organizations identified pairs of community sites that would serve as host sites for their mobile market; these community sites were located in a lower-income and/or low food access community. During the RFP process, final organizations were extensively evaluated by an external committee on several criteria including demonstrated need in the region served by their chosen community sites (20). In total, there were 33 community sites randomized to be an intervention site (*n* = 17 sites) or a planning site (*n* = 16 sites). Intervention sites launched a mobile market shortly after baseline data collection and operated for at least 12 months. The other paired site was assigned to a planning condition that included a year-long community engagement process with the goal of hosting a market or another food access program after the 12-month planning period. Each partner organization completed a memorandum of understanding (MOU) indicating that they will adopt the VV model at participating market sites. In partnership with community sites, partner organizations also agreed to facilitate participant recruitment for the larger VV effectiveness trial; study participants were individuals that frequented community sites and were interested in shopping at a future mobile market. The VV study’s main participant outcomes include F&V consumption, food security, psychosocial factors, and dermal carotenoid concentrations (19); additional details on methods (19) and the results of the effectiveness arm of this study will be reported elsewhere.

### 1.3 Veggie Van Study implementation support

Organizations received support for implementing the VV model through various mediums. Organizations received funding to offset the costs of data collection and implementing the VV model (≤$50,000 per organization). Organizations had discretion over how funds would be allocated between sites. During the selection process for VV study partner organizations, finalists were invited to attend an in-person selection process and training (19, 20). Study partner organizations also had access to an open-access, web-based toolkit informed by the model, known as the VV Toolkit (https://www.mobilemarketcoalition.org/toolkit.html) (19). The VV toolkit was informed by prior research (6, 10, 21) and was developed to enable practitioners to launch a mobile market following the VV model. Partner organizations also received ongoing technical assistance (TA) from the research team. Regular TA engagements provided an opportunity for troubleshooting how to engage customers and receive guidance on how to implement the VV model components. The freqency of TA engagements varied depending on partner availability and needs, but were typically scheduled on a monthly or bi-weekly recurring basis over the intervention period.

The objective of this paper is to describe the implementation arm of the VV study; this extensive process evaluation assessed the implementation of the VV model among study partner organizations. This research aims to fill a gap in literature by providing guidance for designing and conducting process evaluations, particularly for community-based interventions utilizing mixed methods (22, 23).

## 2 Methods and analysis

### 2.1 Overall process evaluation design

**Figure 1:**
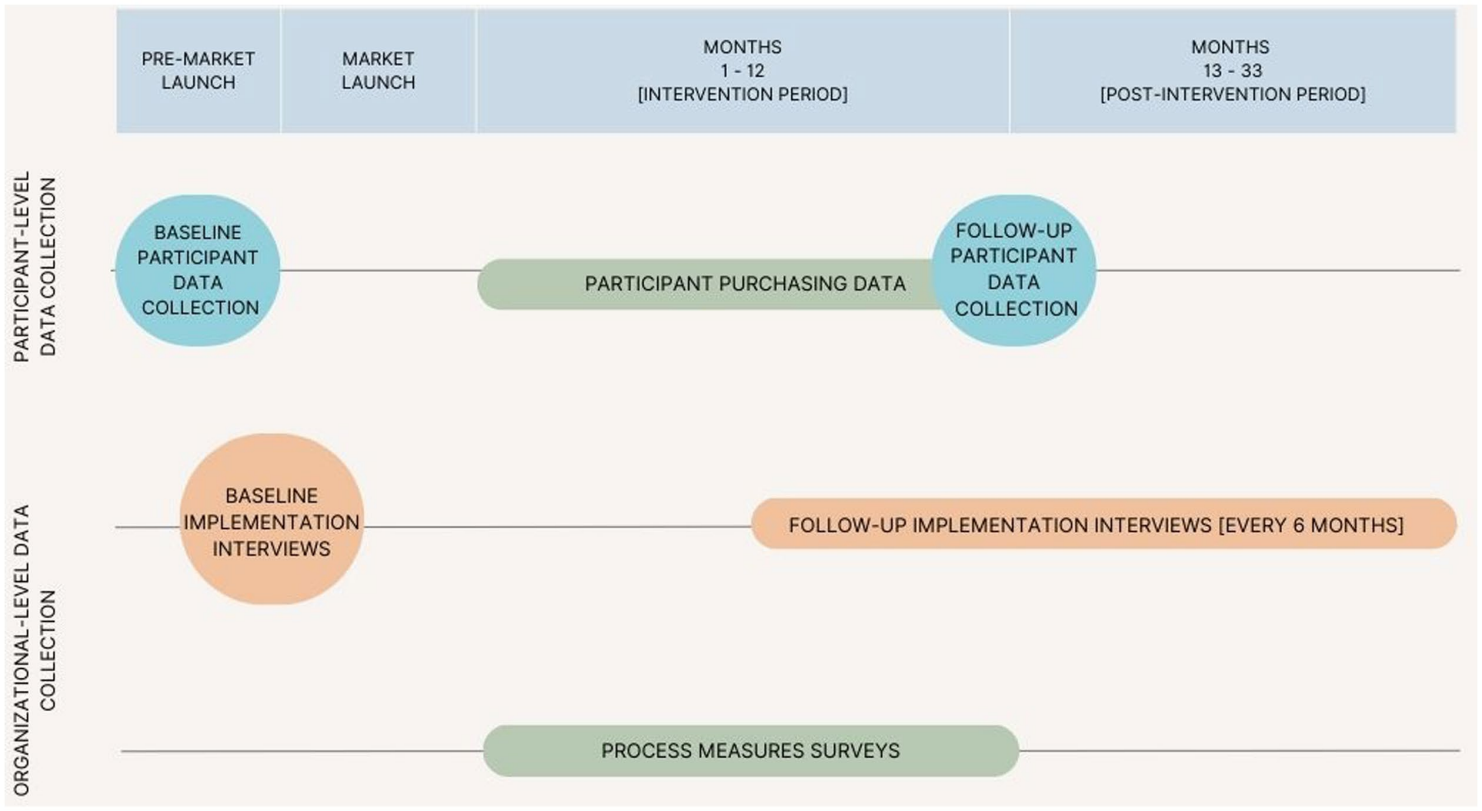
Veggie Van Study data collection timeline.

In addition to facilitating participant-level data collection for the VV study, partner organizations agreed to participate in implementation-related data collection. The design of our process evaluation was threefold: quantitative process measures surveys, qualitative implementation interviews, and purchasing data collected via POS (point-of-sale) at VV mobile market study sites. Figure 1 depicts the timeline for participant and implementation data collection over pre-intervention, intervention, and post-intervention periods. Table 1 presents implementation outcomes and the corresponding data collection activities.

**Table 1:** Process outcomes of the implementation evaluation of the Veggie Van Study.

Process outcome	Definition of process outcomes*	Description	Data collection
Implementation			
Fidelity	The degree to which an intervention was implemented as it was prescribed in the original protocol or as it was intended by the program developers	Organization level:	Process measures surveys
		Adherence to the Veggie Van Model components	
Dose delivered	A component of fidelity; amount of program delivered	Organization level:	Process measures surveys
		The degree to which the Veggie Van Model components were delivered	
Penetration	The integration of a practice within a service setting and its subsystems	Organization level:	Purchasing data
		Percentage of the target population reached by the intervention	
Maintenance			
Sustainability	The extent to which a newly implemented treatment is maintained or institutionalized within a service setting’s ongoing, stable operations	Organization level:	Interviews/process measures surveys
		Integration of the Veggie Van Model into participating study sites and other market sites	
Implementation determinants			
Contextual factors	Domains and constructs that are salient to the process of implementation and interact in complex ways to influence implementation effectiveness	Organization level:	Interviews
		Intervention characteristics	
		Outer setting	
		Inner setting	
		Characteristics of individuals	
		Process	

*Definitions are based on the literature of Proctor et al. (26) and Damschroder et al. (25).

### 2.2 Theoretical framework

The qualitative portion of our process evaluation was informed by the Consolidated Framework for Implementation Research (CFIR); CFIR was developed in 2009 by Damschroder et al. and is comprised of 39 constructs within five major domains (intervention characteristics, outer setting, inner setting, characteristics of individuals, and process) that interact to influence implementation of programs and interventions and their eventual effectiveness (24, 25). CFIR was utilized to construct all implementation interview guides and provided a framework for our data analysis.

For the quantitative portion of our process evaluation, the development of the process measures surveys was rooted in the taxonomy of implementation outcomes set forth by Proctor et al. (26). In response to inconsistencies in the description and measurement of implementation outcomes in implementation research, Proctor et al. established a taxonomy based on a review of the literature to develop a common language (26). This taxonomy outlines the following implementation outcomes: acceptability (satisfaction with the innovation), adoption (innovation uptake), appropriateness (innovation fit), feasibility (innovation utility and suitability), fidelity (adherence), cost, penetration (reach), and sustainability (maintenance) (26). The process measures surveys collect data to assess fidelity which is defined as adherence to the core components of the VV model as they were intended to be delivered (26). In addition, the process measures assessed the dimension of fidelity known as dose or the amount of program delivered (26).

### 2.3 Data collection

#### 2.3.1 Implementation interviews

We conducted in-depth qualitative interviews to understand mobile market operators’ experiences of implementing the VV model. Each organization was asked to identify the individuals who were the most closely engaged with the implementation of the VV model to participate in semi-structured interviews. Up to four representatives from each organization participated in implementation interviews at six time points. Baseline interviews were conducted shortly prior to or just after the launch of new market sites adopting the VV model. We conducted follow-up interviews at 9–12 months, 15 months, 21 months, 27 months, and 33 months post-market launch.

Semi-structured interview guides were informed by the 2009 version of CFIR. We selected the CFIR constructs most salient for inclusion in the interview guide based on findings from our formative work with key informants from mobile market organizations (12). However, constructs in the interview guides were iteratively updated over time based on preliminary findings from earlier implementation interviews. For example, the baseline interview elucidated the need to add constructs such as cosmopolitanism to capture networking activities; in addition, certain constructs were more relevant to later stages of implementation, such as execution. Table 2 presents the CFIR constructs included in the 9–12-month interview guide with example questions. Interview guides were designed to understand the factors in each organization that either enhance or impede VV model implementation. Interviewees were also asked to comment on the technical support and resources (e.g., VV toolkit) provided by the research team and suggest other types of support that would have been helpful. Earlier interviews focused on implementation of the model and expansion to other non-study market sites. Later interviews (27 months and onward) focused on sustainability concerns such as maintenance of the model and costs incurred. When conducting the initial implementation interviews, we found that organizations had feedback and issues related to study activities (e.g., utilizing the POS software, recruiting study participants). Therefore, the 21-month interviews focused on gathering feedback related to the study itself.

**Table 2:** Consolidated framework for implementation research constructs included in 9–12-month follow-up implementation interviews and example questions.

Domains and constructs	Descriptions of constructs	Implementation interview questions
Domain: intervention characteristics		
Relative advantage	Stakeholders’ perception of the advantage of implementing the intervention versus an alternative solution.	What advantages/disadvantages does the Veggie Van model have compared to existing/previous models?
Complexity	Perceived difficulty of implementation, reflected by duration, scope, radicalness, disruptiveness, centrality, and intricacy and number of steps required to implement.	As part of the Veggie Van model, we asked our partners to partner with local organizations that are already serving a similar target market to serve as host sites. How complicated has it been implementing this component of the model?
Design quality & packaging	Perceived excellence in how the intervention is bundled, presented, and assembled.	What is your impression thus far of the helpfulness of the materials you have received for implementing the program from the Veggie Van team? What could be improved to make the materials more helpful?
Cost	Costs of the intervention and costs associated with implementing the intervention including investment, supply, and opportunity costs.	What additional costs, if any, did your organization incur due to offering a produce bundle?
Domain: outer setting		
Customer needs & resources	The extent to which patient needs, as well as barriers and facilitators to meet those needs, are accurately known and prioritized by the organization.	How has implementing the Veggie Van model helped your organization better meet the needs of the individuals you serve (i.e., your customers)?
Cosmopolitanism	The degree to which an organization is networked with other external organizations.	What kind of networking or information exchange do you have with other mobile market organizations, either related to the Veggie Van model, or more generally?
Peer pressure	Mimetic or competitive pressure to implement an intervention; typically because most or other key peer or competing organizations have already implemented or are in a bid for a competitive edge.	To what extent do you think other mobile market organizations are implementing the Veggie Van model?
External policy & incentives	A broad construct that includes external strategies to spread interventions, including policy and regulations (governmental or other central entity), external mandates, recommendations and guidelines, pay-for-performance, collaboratives, and public or benchmark reporting.	What local policies or regulations have made it difficult to implement the Veggie Van model?
Domain: inner setting		
Structural characteristics	The social architecture, age, maturity, and size of an organization.	What are some of the ways that the structure of your organization has influenced the adoption of the Veggie Van model for your mobile market?
Networks & communications	The nature and quality of webs of social networks and the nature and quality of formal and informal communications within an organization.	Can you describe how decisions surrounding the Veggie Van model are communicated in your organization?
Implementation climate	The absorptive capacity for change, shared receptivity of involved individuals to an intervention, and the extent to which use of that intervention will be rewarded, supported, and expected within their organization.	On a scale of 1 to 10, with 1 meaning not enthusiastic at all and 10 meaning very enthusiastic, how would you describe the general level of receptivity in your organization to implement the Veggie Van model? Why did you choose this number?
Implementation climate: tension for change	The degree to which stakeholders perceive the current situation as intolerable or needing change.	What might be some challenges or sources of tension within your organization in terms of implementing the Veggie Van model?
Implementation climate: compatibility	The degree of tangible fit between meaning and values attached to the intervention by involved individuals, how those align with individuals’ own norms, values, and perceived risks and needs, and how the intervention fits with existing workflows and systems.	How well does the Veggie Van model fit with you exiting programs and/or mobile market operations?
Implementation climate: relative priority	Individuals’ shared perception of the importance of the implementation within the organization.	What is the priority of implementing the Veggie Van model relative to other initiatives that are happening now?
Implementation climate: organizational incentives & rewards	Extrinsic incentives such as goal-sharing awards, performance reviews, promotions, and raises in salary, and less tangible incentives such as increased stature or respect.	What is your motivation for wanting to help ensure implementation of the Veggie Van model is successful?
Implementation climate: goals and feedback	The degree to which goals are clearly communicated, acted upon, and fed back to staff, and alignment of that feedback with goals.	How has your organization monitored/assessed progress towards implementation of the Veggie Van and related goals?
Readiness for implementation: leadership engagement	Commitment, involvement, and accountability of leaders and managers with the implementation.	How has leadership within your organization endorsed or supported the implementation of the Veggie Van model?
Readiness for implementation: access to knowledge & information	Ease of access to digestible information and knowledge about the intervention and how to incorporate it into work tasks.	Now I’m going to list some Veggie Van trainings and/or materials that you may have participated in or received. Please provide feedback on each of the resources you have had access to.
Domain: characteristics of individuals		
Knowledge & beliefs about the intervention	Individuals’ attitudes toward and value placed on the intervention as well as familiarity with facts, truths, and principles related to the intervention.	Do you think the Veggie Van model is effective at improving access to fresh fruits and vegetables for lower income communities? Why or why not?
Self-efficacy	Individual belief in their own capabilities to execute courses of action to achieve implementation goals.	On a scale of 1 to 10, with 1 being not confident at all and 10 being very confident, please rate how confident you are that you will be able to successfully implement the Veggie Van model? Why did you choose this number?
Domain: process		
Planning	The degree to which a scheme or method of behavior and tasks for implementing an intervention are developed in advance, and the quality of those schemes or methods.	Can you describe the plan for implementing the Veggie Van model?
Engaging: mobile market customers	Attracting and involving appropriate individuals in the implementation and use of the intervention through a combined strategy of social marketing, education, role modeling, training, and other similar activities.	What is your communication strategy for getting the word out about your mobile market in the community, more broadly?
Engaging: opinion leaders	Individuals in an organization who have formal or informal influence on the attitudes and beliefs of their colleagues with respect to implementing the intervention.	Who are the key influential individuals to get on board with the implementation of the Veggie Van model? How have they been involved?
Engaging: formally appointed internal implementation leaders	Individuals from within the organization who have been formally appointed with responsibility for implementing an intervention as coordinator, project manager, team leader, or other similar role.	Who leads the implementation of the Veggie Van model? How have they been involved?
Engaging: champions	Individuals who dedicate themselves to supporting, marketing, and ‘driving through’ an [implementation], overcoming indifference or resistance that the intervention may provoke in an organization.	Other than your contacts at the community/host sites, are there other individuals outside your organization that serve as champions in support of your mobile market? How have they been involved?
Engaging: external change agents	Individuals who are affiliated with an outside entity who formally influence or facilitate intervention decisions in a desirable direction.	In what ways are the community/host sites you are working with helping your organization run a mobile market adopting the Veggie Van model?
Executing	Carrying out or accomplishing the implementation according to plan.	Has the Veggie Van model been implemented according to plan? Why or why not?

Definitions are based on the literature of Damschroder et al. (25).

Most of the interviews included qualitative questions for each CFIR domain and construct that were adapted from the online CFIR guide (27). However, some of the CFIR constructs were adapted as quantitative questions and asked interviewees to respond based on a Likert scale (e.g., “On a scale of 1 to 10, how confident are you in implementing the nutrition education portion of the Veggie Van model?”). Including quantitative questions helped shorten the interviews and allowed comparisons across time-points and interviewees. In addition, the interview guides included quantitative questions on self-efficacy (Likert scale) and on individual traits such as perseverance and passion for long-term goals, measured using the validated grit scale (28). We attempted to interview the same representatives across time points, but in the case of staff turnover, organizations were asked to identify alternative staff to interview at follow-up.

All interviews were conducted over the phone, lasted approximately 90 min, and were moderated by a researcher trained in qualitative interviewing. Representatives from mobile market organizations were independently interviewed. Each partner organization received compensation for participating in the larger RCT study and interviews were completed as a part of interviewees’ job function, thus they did not receive additional compensation. Some representatives completed a baseline interview but left the study partner organization before follow-up. Those individuals were contacted and invited to participate in 90-min follow-up interviews; those that agreed to participate in a follow-up interview, received a $75 gift card for each interview. The process measures survey is available under Supplementary materials.

#### 2.3.2 Process measures surveys

The goal of the process measures surveys was to understand to what degree the VV model was being implemented at market sites, or fidelity to the model, including the dose of the intervention delivered. The process measures survey is a secure web-based (REDCap) form that mobile market staff can complete in about 15 min each month. The survey is designed to determine how closely organizations adhered to the VV model; survey questions assess whether core components of the VV model (i.e., bundling, nutrition education; etc.) were implemented in a given month and to what degree (i.e., frequency, duration). In addition, the survey measures other important implementation factors including adaptations, interruptions to holding a market (e.g., weather, illness), and staffing availability. Table 3 presents each VV model component and examples of survey questions. Survey questions were iteratively added or amended over the span of the study due to (1) adaptations to the model or market operations that were repeatedly reported, (2) misinterpretation of questions, and (3) to more accurately capture fidelity to the VV model such as the dose of different model components.

**Table 3:** Process measures survey example questions.

VV model core component	Process measures survey questions
Convenient location	“How many times was a mobile market program held at [site name] in the last calendar month?”
	“When the market was held last month, was it always located directly at [site name]?
High quality fresh produce	Where do you most often get produce for this [site name] last month?
	“About what percentage of the produce offered at the market last month was locally sourced?”
Reduced cost payment model	“What type of pricing model are you offering at this market site?”
	“Does this market site accept SNAP for payment?”
	“What types of incentives are accepted at this market site?”
Nutrition education	“Was a food or nutrition lesson/activity offered at the market at [site name] at least once last month?”
	“How often was a nutrition lesson offered at the market?”
Produce bundles	“Was a bundle offered at the market last month?”
	“How many days did the market offer a bundle last month?”

A designated representative with knowledge of mobile market operations at each organization was sent the survey via email around the first of each month to be completed based on the prior month’s mobile market activities. Automated emails were sent throughout the month to remind them to complete the survey. Surveys were sent monthly throughout the course of the intervention (12 months) and were regularly checked for completion and flagged for issues by a research assistant. Baseline and follow-up implementation interview guides are available under Supplementary materials.

#### 2.3.3 Mobile market purchasing data

The goal of collecting individual purchasing data was to assess the penetration or reach of the intervention within each organization’s target community. Partner organizations were each provided with an iPad and a custom iPad-based POS software known as Farmers Register © (Perigee Labs, Inc.). This POS software streamlines mobile market transactions and sales tracking. Mobile market staff received training on Farmers Register © directly from the software developer. Organizations were instructed to utilize the software to process the transactions of all customers (study participants and non-study participants) who shopped at participating market sites implementing the VV model. Purchasing data is transmitted to a data portal that can be accessed by the research team and includes customers’ loyalty ID number, itemized transaction data including sales amounts, product purchased, and forms of tender used.

### 2.4 Data management and analysis

#### 2.4.1 Implementation interviews

Interviews were recorded, transcribed, and checked for accuracy. Qualitative data analysis was completed using the software ATLAS.ti version 8.0. Our approach for our analysis is modeled after the CFIR-driven protocol that Norman et al. (29) applied to their evaluation of the Healthy School Start program. Baseline and follow-up interview data were coded by two independent coders— the lead author (CK) and a research assistant coder analyzed all interview transcripts separately. The first phase of coding involved inductive thematic analysis utilizing open or free coding to identify barriers and facilitators to implementation. The creation and iterative refinement of a codebook throughout the coding process allowed for themes across codes to be combined into subcategories (e.g., funding and resources) within the overarching categories of barriers and facilitators to implementation (25). All coded baseline and follow-up data were merged into one project bundle and the coders met to reconcile coding differences. Regular meetings between coders involved discussion and updates to the codebook throughout the coding process. Coding of baseline interviews was completed first and the codebook generated from that coding process was utilized for subsequent coding of follow-up interviews. Continual updating and refinements occurred during the coding of follow-up data. A second phase of coding involved a deductive organization of interview data into the five domains of CFIR (e.g., inner setting). Reports and queries were generated for facilitators and barriers for each CFIR construct across all partner organizations. Memos were written to summarize each code report and query.

Quantitative interview responses were analyzed using Microsoft Excel for the purposes of descriptive statistics as the sample size was too small to justify the calculations of inferential statistics. Means within and across all organizations were calculated for CFIR Likert responses, self-efficacy scores, and grit scores.

#### 2.4.2 Process measures surveys

The launch of market sites was staggered within and across partners’ market sites to conserve the capacity of the research team while also accommodating partners’ timelines. Therefore, survey data was periodically extracted and cleaned over the duration of the study. This allowed for updates to the survey to be made iteratively based on issues that arose earlier in the study and allowed us to triangulate data from our qualitative data (i.e., implementation interviews) and/or have a member of a research team clarify responses directly with study partners.

To aggregate and quantify the data to assess implementation of individual VV model components as well as the model as a whole, we developed an implementation scoring system. This scoring system allows for comparison of implementation over time, within and between partner organizations. Our approach was modeled after past research in the education field that has developed a rubric which awards points based on fidelity to the core tenets of an intervention and creates a resulting score (30). Scores are initially calculated by site on a monthly basis based on the fidelity rubric; monthly scores for a site are averaged across operating months over the 12-month intervention period and added to the annual site score to create a total implementation score for each site. Total implementation scores are averaged across a partner’s sites to create an overall partner implementation score. Table 4 presents additional details on this rubric and scoring system. The criteria for the scoring rubric were informed by the research team’s knowledge of how the VV model was implemented in the initial pilot and efficacy studies, but there was no pre-established optimal dosage for each VV model component (e.g., frequency of nutrition education). Therefore, criteria for dosage of the components and the corresponding points awarded were guided by discussion among the PI and research team and informed by experience working with mobile market practitioners and understanding what practices are feasible. In addition to yes/no questions, for some components whose implementation may have varied throughout the month (e.g., a market offered a bundle two weeks out of the month), we also collected frequency data in order to quantify the dose delivered of those components.

**Table 4:** Veggie Van model implementation scoring criteria.

VV model component	Monthly implementation score criteria	Annual implementation score criteria	Total implementation score
Convenient location	Did the organization remain at the host site the entire time or relocate to a nearby location serving the same community? [Yes: 5 pts; No: 0 pts]	How long was the operating season? [10–12 months: 5 pts; 8–9 months: 4 pts; 6–7 months: 3 pts; less than 6 months: 0 pts]	
	Did the organization operate at least 3 times per month in operating months? [Yes: 5 pts; No: 0 pts]		
Nutrition education	Implemented some form of food lesson and/or demonstration? [Yes: 5 pts; No: 0 pts]		
	Frequency of implementing food lessons and/or demonstration? [At least bi-weekly (2 or more times): 5 pts; monthly (1 time per month): 3 pts; Never: 0 pts]		
Bundling	Implemented a bundle with an incentive? [Yes: 5 pts; Yes, but without an incentive: 3 pts; No: 0 pts]		
	Frequency of implementing a bundle? [Weekly (3 or more times): 5 pts; bi-weekly: 3 pts; less than 2 times per month: 0 pts]		
Pricing model	Implementing a reduced cost pay model? [Yes: 5 pts; No: 0 pts]	Participate in the SNAP Program? [Yes: 5 pts; No: 0 pts]	
		Participate in at least one incentive program? [*at any point over the course of the year - to account of interruptions in incentive programming that are out of their control*] [Yes: 5 pts; No: 0 pts]	
High quality produce procurement	Procuring high quality produce? [At least some local AND *no* rescued food: 5 pts; At least some local AND *some* rescued food: 3 pts; No local AND/OR *exclusively* rescued food: 0 pts]		
Maximum possible score	40	15	
	[Average of Site’s Monthly Implementation Scores] + [Annual Implementation Score] = Total Implementation Score		55

We will calculate monthly, annual, and total implementation scores for each site in Excel and SAS. For partners that have implemented the VV model across multiple market sites, an overall implementation score will be calculated through averaging the total implementation scores for each site. Additional descriptive statistics will be calculated at the site and partner levels, such as average monthly score for individual VV model components within and across partners. To aid in interpretation of total implementation scores, we will establish thresholds for implementation effectiveness by establishing tertiles (low, transitional, or high implementation) centered around the mean total implementation score and spaced according to Z-scores. Given that implementation scores are at the partner level, our sample size will be small; as such we expect that our analysis will be largely descriptive in nature. However, we plan to combine implementation data with participant dietary data to permit more extensive analyses; these methods will be reported elsewhere. Lastly, our analysis will initially focus on the 12-month intervention period to provide context for interpreting our participant outcomes; however, we will analyze process measures data beyond the 12-month intervention period to assess the maintenance of the VV model at participating study sites.

#### 2.4.3 Mobile market purchasing data

Farmers Register © purchasing data was aggregated at the site and partner level. Variables include number of customers, transactions, amount spent, and most frequent forms of tender used. Penetration will be calculated as a proportion, the percentage of the target population reached by the intervention, by dividing the number of customers served by participating mobile market sites over the potential number of people served in the geographic area. The potential number of people will be obtained through Census data, and the geographic area will be defined as within one mile from the mobile market in urban communities and within 10 miles in rural communities.

## 3 Trial status

At the time of submission of this protocol (December 2025), the VV study completed all data collection (participant and implementation) and concluded delivery of the intervention for all participating sites. All participant data related to our main dietary outcomes have been analyzed and a resulting manuscript is forthcoming. All process measures survey data have been cleaned and scored for the initial 12-month study period; process measures data collected after 12 months are currently being cleaned and scored. All purchasing data has been cleaned and is currently being prepared for analysis. All implementation interview data (*n* = 86 interviews across 10 organizations) have been cleaned, and the majority of the interviews have been analyzed, with the remaining interviews expected to be analyzed within the next 6 months.

## 4 Discussion

This protocol paper details the design of our process evaluation within a multi-state RCT, evaluating the effectiveness of a community-based mobile market intervention. In our main outcome analyses, we did not observe a statistically significant difference between groups for F&V intake or food security (31). Our preliminary implementation data from this process evaluation indicate that implementation of the VV model was greatly hindered by factors such as COVID-19 restrictions, limited organizational capacity, lack of available resources, and perceptions of certain VV model components being complex. Therefore, a robust process evaluation will allow us to determine if the lack of significant findings in dietary outcomes can be explained by subpar implementation and help to understand how to improve implementation in the future.

### 4.1 Strengths and weaknesses

Our process evaluation has several strengths. We were able to collect a large breadth of implementation data over a period of at least 12 months with a diverse set of partner organizations across different regions of the US. Through our process measures data and implementation scoring system, we will be able to assess implementation at the MM site level and monthly which will allow us to understand variations in implementation over time, within, and between organizations. Further, the detailed design of our process measures survey and scoring system will allow us to assess implementation of the model as a whole as well as individual model components. This will enable more sophisticated analyses that will further our understanding of which model components matter the most in terms of how implementation impacts dietary outcomes.

Our extensive implementation interviews allow us to understand contextual factors that explain implementation and enable us to triangulate process measures survey data to ensure accuracy of responses. Our inclusion of a baseline interview heeds past recommendations to utilize CFIR pre-implementation (24); conducting interviews early in the process elucidated that many partner organizations did not fully understand the tenets of the VV model. During the baseline interview, it became apparent that there was confusion about the specific VV model components due to an emphasis on preparing for study activities (e.g., individual-level recruitment). These insights informed ongoing TA services as well as updates to implementation interview guides to better delineate VV model components from study activities. Future iterations of CFIR should expand the innovation domain to include a construct that assesses implementers’ understanding of the intervention. Due to conducting baseline interviews, we were also able to adjust the focus of one of the planned implementation interviews to be solely on partners’ experience working with a university for a research study. Although asking interview questions based on CFIR constructs prior to or around the start of implementation was valuable, responses to questions that assessed constructs such as complexity and adaptability of the innovation were often speculative as implementers may not have had enough experience implementing the VV model to provide an accurate response. Researchers considering utilizing CFIR as a framework for pre-implementation data collection may want to focus more on other domains. Lastly, the inclusion of purchasing data will allow us to quantify the penetration of the intervention, beyond study participants, which is recognized as an understudied implementation outcome (32).

We had a high rate of completion of process measures surveys and implementation interviews for the initial 12-month period. All nine organizations completed at least two implementation interviews, including baseline and the first follow-up. All but one organization completed three implementation interviews during the initial 12-month study period, and five organizations completed all six implementation interviews over the span of about three years. Our original protocol for the implementation evaluation planned for three time-points (baseline, 6 months, and 12 months). However, the opportunity to continue to collect implementation data beyond 12 months arose due to receipt of additional study funding and as a result of organizations launching multiple mobile markets over several years. Therefore, we were able to assess longer-term maintenance of the VV model among most partner organizations. Among the 17 market sites implementing the VV model across nine organizations, sites completed an average of 11 months of process measure survey data during the main study period (range 5–12 months). Eleven sites across seven organizations had complete survey data for all 12 months. Missingness for process measures data was minimal and occurred mainly during the second year of data collection (12–24 months post market launch). Most partners utilized Farmers Register © software throughout the main study period; the organizations that decided to discontinue using the POS software did so due to challenges with the software that were cited in implementation interviews. We attribute our high response rate to having strong relationships with partners as a result of hosting an in-person selection process and training as well as having regular (monthly or bi-weekly) check-in meetings with partners throughout the study period. Partner organizations were assigned to a member of the research team that was available to answer questions as they arose, via email or regular check-in meetings. We also had at least one designated member of the research team coordinating all scheduling for surveys and interviews, data collection, and monitoring missingness.

A limitation of our process evaluation is that baseline interviews took place shortly after market launch for some organizations which is not standard procedure for baseline data collection. This was due to more pressing activities, such as establishing contracts with each partner organization and planning for mobile market launches, that delayed baseline interviews. In addition, due to the aforementioned confusion between the VV model and study activities, the baseline interviews provided less data that was relevant to implementation of the VV model in comparison to follow-up interview data. We also identified several areas of the process measures survey that were interpreted differently between organizations; we recognized that there is a varied lexicon within the mobile market space that needs to be considered in developing survey instruments. For example, our original process measures survey listed “donation-based” as an option for the question asking organizations about the type of payment model implemented. Our team intended for this option to indicate that the organization would be giving customers free produce; whereas some organizations interpreted this option as “pay-what-you-can” model in which customers donate what they can for their produce purchase. In addition, nutrition education was broadly defined in the original process measures survey; therefore, organizations would indicate that they implemented nutrition education when handing out recipe cards which does not quality as delivering a nutrition lesson in the current fidelity rubric and scoring matrix. The process measures survey was also developed before the COVID-19 pandemic and was not designed to account for pandemic adaptations such as virtual nutrition education and produce delivery. We iteratively updated our survey as we learned of these adaptations through our interviews; however, our process measures results may not fully capture activities that were not able to be reported in prior versions. Pilot testing process evaluation surveys with a sub-group of the intended participants could prevent these issues in the future.

We are also limited in the inferences we can make from the analysis of purchasing data. Partner organizations did not consistently register customers into a loyalty program and/or collect demographic data on customers which precludes our ability to understand whether customers are the intended audience (i.e., limited F&V access) for the intervention. Future process evaluations utilizing purchasing data should involve partner organizations in choosing the preferred POS software and/or provide extensive training to ensure the appropriate data is collected. In addition, we are limited in our choice of denominator for calculating a proportion for assessing the penetration of the intervention. Quantifying the potential number of residents or visitors in a target community presents challenges and defining the geographical scope of the intervention can be subjective. Gaining a better understanding of the sphere of influence (i.e., miles) of a mobile market would allow for a more accurate calculation. Furthermore, additional guidance on quantifying the potential target population size for calculating the penetration of an intervention in nebulous community settings is needed. Lastly, a more holistic process evaluation would include assessment of the extent study participants (i.e., customers) were exposed to the intervention, known as dose received (26, 33). Our data collection tools used to assess the main outcomes of the effectiveness trial (i.e., F&V intake) did not assess this outcome; however, focus groups conducted with participants will provide data on acceptability of the intervention and recommendations for improvements.

## 5 Conclusion

This paper contributes to the literature through providing an example of designing an extensive process evaluation that is conducted in tandem with a complex multi-state intervention and RCT trial. Describing our process fulfills recommendations for transparent reporting of the design, implementation, and analysis of process evaluations (22, 23). Although not a guiding framework during the design process, our process evaluation is aligned with guidance from Medical Research Council guidance that recommends evaluations (1) utilize a combination of methods; (2) collect data at multiple time points to capture change over time; (3) asses fidelity, dose, and reach; and (4) ensure that quantitative and qualitative data are integrated to build upon each other (23).
