# Supplementary material for: Protocol for the process evaluation of a mobile produce market intervention to increase fruit and vegetable consumption in lower-income communities: the Veggie Van Study
Source: Front Public Health. 2026 Apr 15;14:1760383. doi: 10.3389/fpubh.2026.1760383 (PMC13126556; doi:10.3389/fpubh.2026.1760383)
Supplement: Supplementary file 1 [file Supplementary_File_1.docx]

**Baseline Implementation Interview Guide and Phone Script**

# Interview Introduction and Consent

Intro: Script for Completing Telephone Recruitment: Hello, may I please speak with [FIRST AND LAST NAME]? My name is [YOUR NAME] and I’m calling from the Veggie Van Study at the University at Buffalo. You had mentioned this would be a good time to call you to complete your Veggie Van interview. Will this still work for you? *[Note to Interviewer: If participant says that they would like to reschedule, exit the survey and schedule another call with the participant at a time that will work better for them.]*

Thank you for agreeing to participate in this interview. Members of the Veggie Van project team are talking to representatives from partner organizations that have been chosen for the study and will be implementing the Veggie Van model for your current or future mobile market.

1. Talking with you and others will help us understand the readiness and needs of your organization to run a mobile market following the Veggie Van model. For a program to be successful in different settings, it’s important to understand factors that influence implementation throughout the process. We will follow up with you or another representative from your organization in about 6-9 months.
2. Some people will be new to mobile markets and others just new to Veggie Van. We are most interested in learning about the Veggie Van model, but it may also be helpful to hear how this might differ from other mobile market experiences

Do you have any questions so far? Let me give you some more details about the interview process:

1. Today’s interview should last approximately 1 hour. If you need to take a break, we can do so at any time. If you need to end the call early, just let me know and we can reschedule to complete it.
2. We will be taking notes, but we want to make sure that we get everything that you say right. Are you okay with us audio-recording this interview?
3. Before we start, I want you to know that there are no right or wrong answers. You are the expert here, so please share any information that you think might be helpful. If you want to tell me something, but you do not want it recorded, please let me know and I will stop the tape.

Your agreement to participate in the interview indicates your consent to participate in this research. Do you have any questions, or would you like to take some time to think about this before beginning the interview?

If NO: I’m going to start interview now:

**CHARACTERISTICS OF INDIVIDUALS**

**KNOWLEDGE AND BELIEFS ABOUT THE INTERVENTION**

1. Prior to being part of the Veggie Van study, did you or your organization have prior experience with mobile markets?
   1. No prior experience
   2. A little prior experience
   3. Substantial prior experience
2. *If prior experience:* How much would you say you know about the Veggie Van model for running mobile markets?
   1. A lot
   2. A little
   3. Not much
   4. I’m not familiar with the model

**INTERVENTION CHARACTERISTICS**

**COMPLEXITY: VV MODEL COMPONENT (****CONVENIENT LOCATION - COMMUNITY PARTNERSHIPS)**

1. As part of the Veggie Van model, we asked our partners to partner with local organizations that are already serving a similar target market to serve as host sites.
   1. *If prior mobile market experience:* Does this differ from what you were formerly doing*? [If the same as previous model, skip to “Self-efficacy” section]*
   2. *If no prior mobile market experience or this differs from former model:* How complicated do you anticipate it will be to implement this component of the model?

**SELF-EFFICACY: VV MODEL COMPONENT (CONVENIENT LOCATION - COMMUNITY PARTNERSHIPS)**

1. On a scale of 1 to 10 with 1 being the easiest and 10 being the hardest, how easy or hard do you think it will be to partner with host sites for hosting the mobile market? (Probe: What made you choose this number?)
2. What would make it easier for you to implement this?

**COMPLEXITY: VV MODEL COMPONENT (****CONVENIENT LOCATION – REGULAR OPERATIONS)**

1. The Veggie Van model recommends that markets operate on a weekly basis, for 10+ months out of the year.
2. *If prior mobile market experience:* Does this differ from your what you were formerly doing? *[If the same as previous models, skip to “Self-efficacy” section] Previously running year round*
3. *If no prior mobile market experience or this differs from former model:* How complicated do you anticipate it will be to implement this component of the model?

**SELF-EFFICACY: VV MODEL COMPONENT (CONVENIENT LOCATION – REGULAR OPERATIONS)**

1. On a scale of 1 to 10 with 1 being the easiest and 10 being the hardest, how easy or hard do you think it will be to operate for 10 months+ out of the year? (Probe: What made you choose this number?)
2. What would make it easier for you to implement this?

**COMPLEXITY: VV MODEL COMPONENT (HIGH QUALITY PRODUCE PROCUREMENT)**

1. As part of the Veggie Van model, we asked our partners to offer high quality, fresh produce at the mobile market. For example, sourcing directly from farms rather than utilizing mostly rescued food, as well as prioritizing local food as much as possible.
2. *If prior mobile market experience:* Does this differ from your what you were formerly doing? *[If the same as previous model, skip to “Self-efficacy” section]*
3. *If no prior mobile market experience or this differs from former model:* How complicated do you anticipate it will be to implement this component of the model?
4. Tell us about where you plan to source your fresh produce.
5. What are the top three sources for sourcing produce for this site? What percentages do you estimate you are procuring from each of these sources?
   - 1. Rescued/donated food (e.g., local retailers donate leftover produce close to expiration)
     2. Direct from farm (including your organization’s own farms, if applicable)
     3. Farmers’ market
     4. Leftover crops from farmers’ fields (gleaning)
     5. Produce auction
     6. Wholesaler/distributor
     7. Other
6. Do you have any specific standards you follow with regards to the type of produce you purchase? Probe: For example, do you prioritize locally or organic produce?
7. On average, about what percentage of the produce offered at the market last month was locally sourced?

**SELF-EFFICACY: VV MODEL COMPONENT (HIGH QUALITY PRODUCE PROCUREMENT)**

1. On a scale of 1 to 10 with 1 being the easiest and 10 being the hardest, how easy or hard do you think it will be to offer high quality, fresh produce? (Probe: What made you choose this number?)
2. What would make it easier for you to implement this?

**COMPLEXITY: VV MODEL COMPONENT (PRICING MODEL/INCENTIVES)**

1. As part of the Veggie Van model, we are asking our partners to adopt a reduced cost pricing model for lower- income customers.
2. *If prior mobile market experience:* Does this differ from what you were formerly doing? *[If the same as previous models, skip to “Self-efficacy” section]*
3. *If no prior mobile market experience or this differs from former model:* How complicated do you anticipate it will be to implement this component of the model?
4. How would you describe the type of pricing model you implement?
5. Free food distribution (produce is given away)
6. Pay-what-you-can (no price is posted on items and customers pay based on means)
7. Suggested price (suggested prices are posted but they are flexible based on customers’ means)
8. Sliding scale (prices are posted and determined based on customers’ income or participation in benefit programs)
9. Set-price market (prices are pre-set by your organization)
10. Other
11. Tell me about what strategies you will use to reduce costs for lower-income consumers (probe: low prices for all markets/certain markets, SNAP matching or other regional incentive program, your own Loyalty program or rewards for certain customers, Veggie Rx program, giveaways)

**SELF-EFFICACY: VV MODEL COMPONENT (PRICING MODEL/INCENTIVES)**

1. On a scale of 1 to 10 with 1 being the easiest and 10 being the hardest, how easy or hard do you think it will be to incorporate a reduced cost pricing model into mobile market operations? (Probe: What made you choose this number?)
2. What would make it easier for you to implement this?

**COMPLEXITY: VV MODEL COMPONENT (BUNDLING)**

1. The Veggie Van model recommends offering bundles or boxes of produce for sale to customers in addition to offering single pieces of produce.
2. *If prior mobile market experience:* Does this differ from your what you were formerly doing? *[If the same as previous models, skip to “Self-efficacy” section]*
3. *If no prior mobile market experience or this differs from former model:* How complicated do you anticipate it will be to implement this component of the model?

**SELF-EFFICACY: VV MODEL COMPONENT (BUNDLING)**

1. On a scale of 1 to 10, with 1 being the easiest and 10 being the hardest, how easy or hard do you think it will be to incorporate bundling into your mobile market operations? (Probe: What made you choose this number?)
2. What would make it easier for you to implement this?

**COMPLEXITY: VV MODEL COMPONENT (NUTRITION EDUCATION)**

1. The Veggie Van model recommends offering food or nutrition education at least bi-weekly.
2. *If prior mobile market experience:* Does this differ from your what you were formerly doing? *[If the same as previous models, skip to “Self-efficacy” section]*
3. *If no prior mobile market experience or this differs from former model:* How complicated do you anticipate it will be to implement this component of the model?

**SELF-EFFICACY: VV MODEL COMPONENT (NUTRITION EDUCATION)**

1. On a scale of 1 to 10 with 1 being the easiest and 10 being the hardest, how easy or hard do you think it will be to incorporate cooking and nutrition education into your mobile market operations? (Probe: What made you choose this number?)
2. What would make it easier for you to implement this?

**CHARACTERISTICS OF INDIVIDUALS**

## **SELF-EFFICACY: model as a whole**

Now I want you to think about all of the components of the Veggie Van model together

1. Overall, on a scale of 1 to 10, with 1 being the easiest and 10 being the hardest, how easy or hard do you think it will be to follow the Veggie Van model? (Probe: What made you choose this number?)

# **Intervention Characteristics**

**RELATIVE ADVANTAGE**

1. *If prior mobile market experience:* From what you know of the Veggie Van model so far, would you say the model is overall relatively similar or different from your prior mobile market experience?
2. Relatively similar
3. Different. How does it differ? _________________________________________
4. What advantages does the Veggie Van model have compared to existing/previous models?
5. What disadvantages does the Veggie Van model have compared to existing/previous models?
6. Did your organization consider any other mobile market models?
   1. Can you describe that mobile market model?
   2. Why would people prefer the alternative?

**ADAPTABILITY**

- 1. What kinds of changes or alterations do you think you will need to make to the Veggie Van model so it will work effectively for your organization?
  2. Do you think you will be able to make these changes? Why or why not?
  3. Who will decide (or what is the process for deciding) whether changes are needed to be made to the Veggie Van model so that it works well in your setting?
  4. How will you know if it is appropriate to make any changes?

**COST**

1. Do you anticipate that following the Veggie Van model will lead to any changes in your program costs? *[Note to interviewer: Interviewee should think about how much the program costs to run regardless of what funding sources they have available (e.g. Veggie Van grant funding)]*
2. No, it will cost about the same to run as our current markets
3. Yes, it will cost more to run than our current markets
4. Yes, it will cost less to run than our current market
5. If yes, what are the biggest contributors to the cost differences?

**COMPLEXITY: RESEARCH ACTIVITIES**

1. As part of the Veggie Van study, we asked our partners to use the Farmers Register Point-of-Sale System.
2. *If prior mobile market experience:* Does this differ from your what you were formerly doing? *[If the same as previous model, skip to “Self-efficacy” section]*
3. *If no prior mobile market experience or this differs from former model:* How complicated do you anticipate it will be to implement this?

**SELF-EFFICACY: RESEARCH ACTIVITIES**

1. On a scale of 1 to 10 with 1 being the easiest and 10 being the hardest, how easy or hard do you think it will be to incorporate Farmers Register Point-of Sale into mobile market operations? (Probe: What made you choose this number?)
2. What would make it easier for you to implement this?

**COMPLEXITY: RESEARCH ACTIVITIES**

1. As part of the Veggie Van study, we asked our partners to collect demographic information from customers using the Farmer’s Register software.
2. *If prior mobile market experience:* Does this differ from your what you were formerly doing? *[If the same as previous model, skip to “Self-efficacy” section]*
3. *If no prior mobile market experience or this differs from former model:*  How complicated do you anticipate it will be to implement this?

**SELF-EFFICACY: RESEARCH ACTIVITIES**

1. On a scale of 1 to 10 with 1 being the easiest and 10 being the hardest, how easy or hard do you think it will be to collect demographic information using Farmers Register? (Probe: What made you choose this number?)
2. What would make it easier for you to implement this?

# **INNER SETTING**

## **Implementation Climate**

1. On a scale of 1 to 10, with 1 meaning not enthusiastic at all and 10 meaning very enthusiastic, how would you describe the general level of receptivity in your organization to implementing the Veggie Van model? (Probe: What made you choose this number?) (Probe: What made you choose this number?)

**TENSION FOR CHANGE**

1. On a scale of 1 to 10, with 1 meaning not worried at all and 10 meaning very worried, how would you describe the general concern in your organization about meeting the recommendations provided by the Veggie Van model? (Probe: What made you choose this number?)
2. On the same scale, how concerned is your organization overall about the costs associated with implementing the Veggie Van model? (Probe: What made you choose this number?)
3. What might be some challenges or sources of tension within your organization in terms of implementing the Veggie Van model?

# **Outer Setting**

## **Customer Needs and Resources**

1. How do you think implementing the Veggie Van model will help your organization better meet the needs of the individuals you serve (i.e. your customers)?
2. Do you anticipate having to alter the model in order to better meet the needs and preferences of the individuals served by your organization?
3. Do you think implementing the Veggie Van model will introduce any new barriers (compared to how you were running your market before) for the individuals you serve (i.e. your customers)?

## **External Policies and Incentives**

1. What local policies or regulations may make it difficult to implement the Veggie Van model?
   1. If not mentioned, probe on the following:
      1. Zoning restrictions
      2. Parking limitations
      3. Food safety regulations
      4. Signing up for nutrition assistance or incentive programs (e.g. SNAP)
      5. Are there any other policies or regulations that I didn’t mention that you think may be a barrier to implementing the Veggie Van model?
   2. *If currently operating*: Are there any of the barriers you mentioned specific to following the Veggie Van model, or have you faced similar issues when running your mobile market?

# **Inner Setting**

## **Structural Characteristics**

1. What are some of the ways that the structure of your organization will influence the adoption of the Veggie Van model for your mobile market?
   1. For the following statements related to organizational structure, tell me how strongly you agree or disagree with each. (strongly agree, agree, neither agree nor disagree, disagree, strongly disagree)
      1. *My organization has enough experience with mobile markets to implement the Veggie Van model*
      2. *My organization has enough staff to implement the Veggie Van model*
      3. *My organization has enough experience with taste testing/cooking demos to implement the Veggie Van model*
      4. *My organization has enough experience with nutrition education to implement the Veggie Van model*
      5. *My organization has enough Level of experience with data collection to implement the Veggie Van model*
      6. *My organization’s vision and mission align with the Veggie Van model SA*
      7. *My organization has enough support from Board of Directors/Advisory Board to implement the Veggie Van model*
      8. *My organization has enough support from supportive local government to implement the Veggie Van model*
      9. *My organization has enough funding to implement the Veggie Van model SA*
      10. *My organization has enough resources available, other than staff (e.g. office space, marketing materials), to implement the Veggie Van model*
      11. *Anything else? _______________________*
2. What kinds of infrastructure changes to your organization will be needed to assist with implementing the Veggie Van model?
   1. *Interviewer to note answers from list below and probe on the following as time allows (may skip probes based on timing):*
      1. *Hire more staff*
      2. *Staff training on taste testing/cooking demos*
      3. *Staff training on nutrition education*
      4. *Staff training on data collection*
      5. *Secure funding*
      6. *Secure resources, other than staff (e.g. office space, marketing materials)*
      7. *Anything else? _______________________*

**READINESS FOR IMPLEMENTATION: AVAILABLE RESOURCES**

1. Do you expect to have sufficient resources to implement and administer the Veggie Van model over the study period?
2. What resources are you counting on?
3. What resources will not be available that you will need?
4. What would make it easier for you to implement the Veggie Van model?
5. What other resources would you need?
6. How do you expect to secure necessary resources?
7. What information, in addition to study measures that we have asked you to collect, would you like to collect as you implement the Veggie Van model?
8. What is your plan for collecting this information as you implement the Veggie Van model?

**GOALS & FEEDBACK**

1. Have you/your organization set goals related to the implementation of the Veggie Van model?
   1. How will your organization assess progress towards implementation of the Veggie Van and related goals?
   2. Will you or your organization be monitoring the implementation of the Veggie Van model?

## **Relative Priority**

1. What kinds of high-priority initiatives or activities (i.e. top three priorities) are already happening at your organization?
2. What is the priority of getting the Veggie Van model implemented relative to other initiatives that are happening now?
3. Will the implementation conflict with these priorities?
4. Will the implementation help achieve (or relieve pressure related to) these priorities?

## **Readiness for Implementation: Leadership Engagement**

*Ask questions to anyone other than the executive director of an organization (confirm organizational role as needed)*

1. How has leadership within your organization endorsed or supported the implementation of the Veggie Van model?
   1. What level of endorsement or support have you seen or heard?
2. How has leadership at your organization been involved with implementing the Veggie Van model so far? If so, to what extent?
3. Yes, they know about our intentions to implement the Veggie Van model, but are not involved in the implementation process
4. Yes, they know about our intentions to implement the Veggie Van model and are involved in the implementation process
5. No, they do not know about our intentions to implement the Veggie Van model and are not involved in the implementation process
6. *Anything else? _______________________*
7. What kind of support or actions can you expect from leaders in your organization to help make implementation successful?
   1. *Interviewer to note answers from list below and probe on the following as time allows (may skip probes based on timing):*
      1. *Financial (e.g. grant funds)*
      2. *Staffing - administrative*
      3. *Staffing – logistics/operations*
      4. *Strategic planning and problem solving*
      5. *Marketing and outreach resources*
      6. *Procurement and sourcing resources*
      7. *Navigating regulations and policy*
      8. *Anything else? _______________________*

### **Readiness for Implementation: Access to Knowledge and Information**

1. What training have you received from the Veggie Van team on implementing the Veggie Van model? Examples could include webinars, the mobile market summit, one-on-one meetings; etc.
   1. Please provide feedback on the training you have received.

# **Intervention Characteristics**

## **Design Quality, Materials, and Training**

1. On a scale of 1 to 10, with 1 meaning not helpful and 10 meaning very helpful, what is your impression thus far of the helpfulness of the materials (e.g., Veggie Van toolkit) you have received for implementing the Veggie Van model from the Veggie Van team? (Probe: What made you choose this number?)
   1. What could be improved to make the materials more helpful? _______________________
2. On the same scale of 1 to 10, with 1 meaning not helpful and 10 meaning very helpful, what is your impression thus far of the helpfulness of the training for implementing the Veggie Van model that you have received from the Veggie Van team? (Probe: What made you choose this number?)
   1. What could be improved to make the training more helpful? _______________________

# **Characteristics of Individuals**

## **SELF-EFFICACY**

1. On a scale of 1 to 10, with 1 being not confident at all and 10 being very confident, please rate how confident you are that you will be able to successfully implement the Veggie Van model? (Probe: What made you choose this number?)
2. On a scale of 1 to 10, with 1 being not confident at all and 10 being very confident, how confident do you think your colleagues in your organization feel about implementing the Veggie Van model? (Probe: What made you choose this number?)

## **OTHER PERSONAL ATTRIBUTES**

The next set of questions are focused on you so we can gain a better understanding of what type of teams are best for running mobile markets.

1. How many years have you been working at *[organization]*?
2. What is your current position?
   1. Administration
   2. Market manager
   3. Market staff
   4. Evaluation team
   5. Other: ____________________
3. What is the highest level of education you have completed?
4. Eighth grade or less
5. Some high school
6. High school graduate or GED
7. Trade or beauty school graduate
8. Some college
9. College graduate
10. More than undergraduate (some post graduate, post graduate, or professional degree)
11. Before working with the mobile market, did you have any training or education related to your program?
12. *Interviewer to probe on the following after respondent has answered unprompted:*
13. *Public Health*
14. *Nutrition*
15. *Business management*
16. *Cooking*
17. *Agriculture*
18. *Marketing or Communications*
19. *Community Engagement*
20. How would you describe your relationship with the communities served by your mobile market? *[Note to interviewer: do not read answers, but may confirm which they fall into]*
21. *I consider myself a member of the target communities (select one or more of the below answers)*
    - 1. *Lives in the same neighborhood*
      2. *Similar economic background*
      3. *Same race or ethnicity*
22. *I am not a member of the target communities, but I have been working with them for a long time and am trusted by community members*
23. *I do not consider myself a member of the target community*

**GRIT SCALE**

1. Now I’m going to ask you to tell me a bit about your personality. Please feel free to tell me to skip any questions you don’t feel comfortable answering. For the following personality traits, please tell me if you feel the statement is: very much like me, mostly like me, somewhat like me, not much like me, or not like me at all.
2. New ideas and projects sometimes distract me from previous ones
   - 1. *Very much like me*
     2. *Mostly like me*
     3. *Somewhat like me*
     4. *Not much like me*
     5. *Not like me at all*
3. Setbacks don’t discourage me
   - 1. *Very much like me*
     2. *Mostly like me*
     3. *Somewhat like me*
     4. *Not much like me*
     5. *Not like me at all*
4. I have been obsessed with a certain idea or project for a short time but later lost interest
   - 1. *Very much like me*
     2. *Mostly like me*
     3. *Somewhat like me*
     4. *Not much like me*
     5. *Not like me at all*
5. I am a hard worker
6. *Very much like me*
7. *Mostly like me*
8. *Somewhat like me*
9. *Not much like me*
10. *Not like me at all*
11. I often set a goal but later choose to pursue a different one
12. *Very much like me*
13. *Mostly like me*
14. *Somewhat like me*
15. *Not much like me*
16. *Not like me at all*
17. I have difficulty maintaining my focus on projects that take more than a few months to complete
18. *Very much like me*
19. *Mostly like me*
20. *Somewhat like me*
21. *Not much like me*
22. *Not like me at all*
23. I finish whatever I begin
24. *Very much like me*
25. *Mostly like me*
26. *Somewhat like me*
27. *Not much like me*
28. *Not like me at all*
29. I am diligent
30. *Very much like me*
31. *Mostly like me*
32. *Somewhat like me*
33. *Not much like me*
34. *Not like me at all*

**PROCESS**

**PLANNING**

1. Can you describe the plan for implementing the Veggie Van model?
   1. Who is involved in the planning process?

**ENGAGING: EXTERNAL CHANGE AGENTS [HOST SITES]/ KEY STAKEHOLDERS**

1. On a scale of 1 to 10, with 1 being not supportive at all and 10 being extremely supportive, how supportive of your work are the community/host sites you are working with for the Veggie Van study (both market and planning sites)?
   1. In what ways are the community/host sites you are working with helping your organization run a mobile market?

**ENGAGING: CHAMPIONS [OTHER THAN HOST SITES]**

1. Are there other individuals outside your organization who you think will serve as champions in support of your mobile market?
2. In what ways will these individuals support the mobile market?

### **engaging: CUSTOMERS**

1. What is your communication strategy for getting the word out about your mobile market in the community?
   1. *Interviewer to probe on the following after respondent has answered unprompted:*
      1. *Online dissemination (e.g. social media, emails)*
      2. *Paper marketing (e.g. mailers, brochures, flyers)*
      3. *Community meetings and presentations*
      4. *Press coverage (e.g. news, radio)*
      5. *Word of mouth*
2. How will you or your colleagues communicate with current and potential customers, including those that filled out interest forms, about the mobile market?
   1. *Interviewer to probe on the following after respondent has answered unprompted:*
      1. *Online dissemination (e.g. social media, emails)*
      2. *Paper marketing (e.g. mailers, brochures, flyers)*
      3. *Community meetings and presentations*
      4. *Press coverage (e.g. news, radio)*
      5. *Word of mouth*
3. How often do you use the modes of communication we just discussed to connect with your current and potential customers?
   1. Daily
   2. Weekly
   3. Bi-weekly
   4. Monthly
   5. Other: _______________
4. Do you currently or do you plan to have a customer newsletter about the mobile market?
5. Yes
6. No
7. How often will/do you send the customer newsletter about the mobile market?
8. Weekly
9. Bi-weekly
10. Monthly
11. Quarterly
12. How will/do you send the customer newsletter about the mobile market?
13. Electronic
14. Hand out at market
15. Mail to homes
16. Other: ___________________
17. So far, how have the individuals you plan to serve received the mobile market? *[Note to interviewer: do not read answers, but may confirm which they fall into]*
18. The community is aware of the mobile market
19. The community is aware and eager for the mobile market to start
20. The community is aware and concerned about the mobile market
21. The community is largely unaware of the mobile market

# Interview Exit

## *Exit A*

Again, we would like to thank you for your participation in this Veggie Van interview. We may contact you for additional information – would that be ok?

1. Yes
2. No

If you have any questions about the Veggie Van Study, please feel free to contact us by phone at (XXX) XXX-XXXX or via e-mail at XXXXXXXX. We will reach out in approximately 2 months to schedule a 3-month follow-up interview with you or another representative at your organization. Thank you and have a great day!

## *Exit B (If a follow-up call is warranted)*

Again, we would like to thank you for your participation in this Veggie Van interview. We were unable to complete all of our interview questions during this phone call. Would you be willing to schedule an additional phone call to speak with us? *[Note to interviewer: Proceed to scheduling follow-up phone call if the participant is willing. If they are unwilling, conclude phone call]* If you have any questions about the Veggie Van Study, please feel free to contact us by phone at (XXX) XXX-XXXX or via e-mail at XXXXXXXXX. Thank you and have a great day!
