# Supplementary material for: Protocol for the process evaluation of a mobile produce market intervention to increase fruit and vegetable consumption in lower-income communities: the Veggie Van Study
Source: Front Public Health. 2026 Apr 15;14:1760383. doi: 10.3389/fpubh.2026.1760383 (PMC13126556; doi:10.3389/fpubh.2026.1760383)
Supplement: Supplementary file 2 [file Supplementary_File_2.docx]

**9-12 Month Follow-up Implementation Interview Guide and Phone Script**

**Interview Introduction and Consent**

Intro: Script for Completing Telephone Recruitment: Hello, may I please speak with [FIRST AND LAST NAME]? My name is [YOUR NAME] and I’m calling from the Veggie Van Study at the University at Buffalo. You had mentioned this would be a good time to call you to complete your Veggie Van interview. Will this still work for you? *[Note to Interviewer: If participant says that they would like to reschedule, exit the survey and schedule another call with the participant at a time that will work better for them.]*

Thank you for agreeing to participate in this interview. Members of the Veggie Van project team are talking to representatives from partner organizations that have been chosen for the study and will be implementing the Veggie Van model for your current or future mobile market.

1. Talking with you and others will help us understand the readiness and needs of your organization to run a mobile market following the Veggie Van model. For a program to be successful in different settings, it’s important to understand factors that influence implementation throughout the process.
2. Some people will be new to mobile markets and others just new to Veggie Van. We are most interested in learning about how the implementation of the Veggie Van model has been going, but it may also be helpful to hear how this model might differ from other mobile market experiences.

Do you have any questions so far? Let me give you some more details about the interview process:

1. Today’s interview should last approximately 1 hour. If you need to take a break, we can do so at any time. If you need to end the call early, just let me know and we can reschedule to complete it.
2. We will be taking notes, but we want to make sure that we get everything that you say right. Are you okay with us audio-recording this interview?
3. Before we start, I want you to know that there are no right or wrong answers. You are the expert here, so please share any information that you think might be helpful. If you want to tell me something, but you do not want it recorded, please let me know and I will stop the tape.

Your agreement to participate in the interview indicates your consent to participate in this research. Do you have any questions, or would you like to take some time to think about this before beginning the interview?

If NO: I’m going to start interview now:

**CHARACTERISTICS OF INDIVIDUALS**

**KNOWLEDGE AND BELIEFS ABOUT THE INTERVENTION**

1. Prior to being part of the Veggie Van study, did you or your organization have prior experience with mobile markets?
   1. No prior experience
   2. A little prior experience
   3. Substantial prior experience
2. *If prior mobile market experience:* How much would you say you know about the Veggie Van model for running mobile markets?
   1. A lot
   2. A little
   3. Not much
   4. I’m not familiar with the model

**INTERVENTION CHARACTERISTICS**

**COMPLEXITY: VV MODEL COMPONENT (CONVENIENT LOCATION - COMMUNITY PARTNERSHIPS)**

1. As part of the Veggie Van model, we asked our partners to partner with local organizations that are already serving a similar target market to serve as host sites.
   1. *If prior mobile market experience:* Does this differ from what you were formerly doing*? [If the same as previous model, skip to “Self-efficacy” section]*
   2. *If no prior mobile market experience or this differs from former model:* How complicated has it been implementing this component of the model?

**COST: VV MODEL COMPONENT (CONVENIENT LOCATION - COMMUNITY PARTNERSHIPS)**

1. *If this differs from former model:* What additional costs, if any, did your organization incur due to partnering with local partners to host the market?

**SELF-EFFICACY: VV MODEL COMPONENT (CONVENIENT LOCATION - COMMUNITY PARTNERSHIPS)**

1. On a scale of 1 to 10 with 1 being the easiest and 10 being the hardest, how easy or hard do you think this is to partner with host sites for hosting the mobile market? (Probe: What made you choose this number?)
2. What would make it easier for you to implement this?

**COMPLEXITY: VV MODEL COMPONENT (CONVENIENT LOCATION – REGULAR OPERATIONS)**

1. The Veggie Van model recommends that markets operate on a weekly basis, for 10+ months out of the year.
2. *If prior mobile market experience:* Does this differ from what you were formerly doing? *[If the same as previous models, skip to “Self-efficacy” section] Previously running year round*
3. *If no prior mobile market experience or this differs from former model:* How complicated has it been implementing this component of the model?

**COST: VV MODEL COMPONENT (CONVENIENT LOCATION – REGULAR OPERATIONS)**

1. *If this differs from former model:* What additional costs, if any, did your organization incur due to due to operating on a weekly basis, for 10 months out of the year?

**SELF-EFFICACY: VV MODEL COMPONENT (CONVENIENT LOCATION – REGULAR OPERATIONS)**

1. On a scale of 1 to 10 with 1 being the easiest and 10 being the hardest, how easy or hard do you think it is to operate on a weekly basis, for 10 months+ out of the year? (Probe: What made you choose this number?)

**COMPLEXITY: VV MODEL COMPONENT (HIGH QUALITY PRODUCE PROCUREMENT)**

1. As part of the Veggie Van model, we asked our partners to offer high quality, fresh produce at the mobile market. For example, sourcing directly from farms rather than utilizing mostly rescued food, as well as prioritizing local food as much as possible.
2. *If prior mobile market experience:* Does this differ from what you were formerly doing? *[If the same as previous model, skip to “Self-efficacy” section]*
3. *If no prior mobile market experience or this differs from former model:* How complicated has it been implementing this component of the model?
4. Tell us about where you source your fresh produce.
5. What are the top three sources for sourcing produce for this site? What percentages do you estimate you are procuring from each of these sources?
   - 1. Rescued/donated food (e.g., local retailers donate leftover produce close to expiration)
     2. Direct from farm (including your organization’s own farms, if applicable)
     3. Farmers’ market
     4. Leftover crops from farmers’ fields (gleaning)
     5. Produce auction
     6. Wholesaler/distributor
     7. Other
6. Do you have any specific standards you follow with regards to the type of produce you purchase? Probe: For example, do you prioritize locally or organic produce?
7. On average, about what percentage of the produce offered at the market last month was locally sourced?

**COST: VV MODEL COMPONENT (HIGH QUALITY PRODUCE PROCUREMENT)**

1. *If this differs from former model:* What additional costs, if any, did your organization incur due to offering high quality, fresh produce?

**SELF-EFFICACY: VV MODEL COMPONENT (HIGH QUALITY PRODUCE PROCUREMENT)**

1. On a scale of 1 to 10 with 1 being the easiest and 10 being the hardest, how easy or hard do you think it is to offer high quality, fresh produce? (Probe: What made you choose this number?)
   1. What would make it easier for you to implement this?

**COMPLEXITY: VV MODEL COMPONENT (PRICING MODEL/INCENTIVES)**

1. As part of the Veggie Van model, we are asking our partners to adopt a reduced cost pricing model for lower- income customers.
2. *If prior mobile market experience:* Does this differ from what you were formerly doing? *[If the same as previous models, skip to “Self-efficacy” section]*
3. *If no prior mobile market experience or this differs from former model:* How complicated has it been implementing this component of the model?
4. How would you describe the type of pricing model you implement?
   - 1. Free food distribution (produce is given away)
     2. Pay-what-you-can (no price is posted on items and customers pay based on means)
     3. Suggested price (suggested prices are posted but they are flexible based on customers’ means)
     4. Sliding scale (prices are posted and determined based on customers’ income or participation in benefit programs)
     5. Set-price market (prices are pre-set by your organization)
     6. Other
5. Tell me about what strategies you use to reduce cost for lower-income consumers (probe: low prices for all markets/certain markets, SNAP matching or other regional incentive program, your own Loyalty program or rewards for certain customers, Veggie Rx program, giveaways)
   1. For each program/strategy you mentioned, tell me more about how that program works and indicate who is eligible to participate.

**COST: VV MODEL COMPONENT (PRICING MODEL/INCENTIVES)**

1. *If this differs from former model:* What additional costs, if any, did your organization incur due to due to adopting a reduced cost pricing model?

**SELF-EFFICACY: VV MODEL COMPONENT (PRICING MODEL/INCENTIVES)**

1. On a scale of 1 to 10 with 1 being the easiest and 10 being the hardest, how easy or hard do you think it is to incorporate a reduced cost pricing model into mobile market operations? (Probe: What made you choose this number?)
2. What would make it easier for you to implement this?

**COMPLEXITY: VV MODEL COMPONENT (BUNDLING)**

1. The Veggie Van model recommends offering bundles or boxes of produce for sale to customers in addition to offering single pieces of produce.
2. *If prior mobile market experience:* Does this differ from your what you were formerly doing? *[If the same as previous models, skip to “Self-efficacy” section]*
3. *If no prior mobile market experience or this differs from former model:* How complicated has it been implementing this component of the model?

**COST: VV MODEL COMPONENT (BUNDLING)**

1. *If this differs from former model:* What additional costs, if any, did your organization incur due to offering a bundle?

**SELF-EFFICACY: VV MODEL COMPONENT (BUNDLING)**

1. On a scale of 1 to 10, with 1 being the easiest and 10 being the hardest, how easy or hard do you think it is to incorporate bundling into your mobile market operations? (Probe: What made you choose this number?)
   1. What would make it easier for you to implement this?

**COMPLEXITY: VV MODEL COMPONENT (NUTRITION EDUCATION)**

1. The Veggie Van model recommends offering cooking and nutrition education at least 1-2 times per month.
2. *If prior mobile market experience:* Does this differ from your what you were formerly doing? *[If the same as previous models, skip to “Self-efficacy” section]*
3. *If no prior mobile market experience or this differs from former model:* How complicated has it been implementing this component of the model?

**COST: VV MODEL COMPONENT (NUTRITION EDUCATION)**

1. *If this differs from former model:* What additional costs, if any, did your organization incur due to due to operating offering nutrition education?

**SELF-EFFICACY: VV MODEL COMPONENT (NUTRITION EDUCATION)**

1. On a scale of 1 to 10 with 1 being the easiest and 10 being the hardest, how easy or hard do you think it is to incorporate cooking and nutrition education into your mobile market operations? (Probe: What made you choose this number?)
   1. What would make it easier for you to implement this?

**INTERVENTION CHARACTERISTICS**

**RELATIVE ADVANTAGE**

1. *If prior mobile market experience:* From what you know of the Veggie Van model so far, would you say the model is overall relatively similar or different from your prior mobile market experience?
2. Relatively similar
3. Different. How does it differ? _________________________________________
4. *If prior mobile market experience:* How has being part of the Veggie Van study changed your mobile market operations?
5. What advantages does the Veggie Van model have compared to existing/previous models?
6. What disadvantages does the Veggie Van model have compared to existing/previous models?.

**INNER SETTING**

**COMPATIBILITY***

1. How well does the Veggie Van model fit with your existing programs and/or mobile market operations?

**CHARACTERISTICS OF INDIVIDUALS**

## **SELF-EFFICACY: model as a whole**

Now I want you to think about all of the components of the Veggie Van model together

1. Overall, on a scale of 1 to 10, with 1 being the easiest and 10 being the hardest, how easy or hard do you think it is to follow the Veggie Van model? (Probe: What made you choose this number?)

**COMPLEXITY: RESEARCH ACTIVITIES**

1. As part of the Veggie Van study, we asked our partners to use the Farmers Register Point-of-Sale System.
2. *If prior mobile market experience:* Does this differ from what you were formerly doing? *[If the same as previous model, skip to “Self-efficacy” section]*
3. *If no prior mobile market experience or this differs from former model:* How complicated has it been implementing this component of the model?

**COST: RESEARCH ACTIVITIES**

1. *If this differs from former model:* What additional costs, if any, did your organization incur due to due to using the Farmers Register POS?

**SELF-EFFICACY: RESEARCH ACTIVITIES**

1. On a scale of 1 to 10 with 1 being the easiest and 10 being the hardest, how easy or hard do you think it is to incorporate Farmers Register Point-of Sale into mobile market operations? (Probe: What made you choose this number?)
   1. What would make it easier for you to implement this?

**COMPLEXITY: RESEARCH ACTIVITIES**

1. As part of the Veggie Van study, we asked our partners to collect demographic information from customers using the Farmer’s Register software.
2. *If prior mobile market experience:* Does this differ from what you were formerly doing? *[If the same as previous model, skip to “Self-efficacy” section]*
3. *If no prior mobile market experience or this differs from former model:*  How complicated has it been implementing this component of the model? (name, address, email, birthday)

**SELF-EFFICACY: RESEARCH ACTIVITIES**

1. On a scale of 1 to 10 with 1 being the easiest and 10 being the hardest, how easy or hard do you think it is to collect demographic information using Farmers Register? (Probe: What made you choose this number?)
2. What would make it easier for you to implement this?

**DESIGN QUALITY, MATERIALS, AND TRAINING**

1. On a scale of 1 to 10, with 1 meaning not helpful and 10 meaning very helpful, what is your impression thus far of the helpfulness of the materials you have received for implementing the program from the Veggie Van team? (Probe: What made you choose this number?)
2. What could be improved to make the materials more helpful? _______________________
3. On the same scale of 1 to 10, with 1 meaning not helpful and 10 meaning very helpful, what is your impression thus far of the helpfulness of the training for implementing the program that you have received from the Veggie Van team? (Probe: What made you choose this number?)
4. What could be improved to make the training more helpful? _______________________

**CHARACTERISTICS OF INDIVIDUALS**

**KNOWLEDGE AND BELIEFS ABOUT THE INTERVENTION**

1. Do you think the Veggie Van model is effective at improving access to fresh fruits and vegetables for lower income communities?
   1. Why or why not?

**OUTER SETTING**

**CUSTOMER NEEDS AND RESOURCES***

1. How has implementing the Veggie Van model helped your organization better meet the needs of the individuals you serve (i.e. your customers)?
2. Have you altered the model in order to better meet the needs and preferences of the individuals served by your organization?
3. Do you think implementing the Veggie Van model has introduced any new barriers (compared to how you were running your market before) for the individuals you serve (i.e. your customers)?

**COSMOPOLITAN***

1. What kind of networking or information exchange do you have with other mobile market organizations, either related to the Veggie Van model, or more generally?

**PEER PRESSURE**

1. To what extent do you think other mobile market organizations are implementing the Veggie Van model?
   1. How does this effect support for implementing the model with your market?
2. To what extent has implementing the Veggie Van model provided an advantage for your organization compared to other organizations in your area?

**EXTERNAL POLICIES AND INCENTIVES***

1. What local policies or regulations have made it difficult to implement the Veggie Van model?
   1. If not mentioned, probe on the following:
      1. Zoning restrictions
      2. Parking limitations
      3. Food safety regulations
      4. Signing up for nutrition assistance or incentive programs (e.g. SNAP)
      5. Are there any other policies or regulations that I didn’t mention that you think may be a barrier to implementing the Veggie Van model?
   2. *If currently operating*: Are any of the barriers you mentioned specific to following the Veggie Van model, or have you faced similar issues when running your mobile market?

**INNER SETTING**

**IMPLEMENTATION CLIMATE**

1. On a scale of 1 to 10, with 1 meaning not enthusiastic at all and 10 meaning very enthusiastic, how would you describe the general level of receptivity in your organization to implement the Veggie Van model? (Probe: What made you choose this number?)

**NETWORKS AND COMMUNICATIONS***

1. Can you describe how decisions surrounding the Veggie Van model are communicated in your organization?

**RELATIVE PRIORITY**

1. What kinds of high-priority initiatives or activities (i.e. top three priorities) are already happening at your organization?
2. What is the priority of getting the Veggie Van model implemented relative to other initiatives that are happening now?
3. Will the implementation conflict with these priorities?
4. Will the implementation help achieve (or relieve pressure related to) these priorities?

**TENSION FOR CHANGE***

1. On a scale of 1 to 10, with 1 meaning not worried at all and 10 meaning very worried, how would you describe the general concern in your organization about meeting the recommendations provided by the Veggie Van model? For this question we are asking specifically about how the market is run and not the community engagement or data collection that happened before the market started (Probe: What made you choose this number?)
2. On the same scale, how concerned is your organization overall about the costs associated with implementing the Veggie Van model? (Probe: What made you choose this number?)

**STRUCTURAL CHARACTERISTICS***

1. What are some of the ways that the structure of your organization has influenced the adoption of the Veggie Van model for your mobile market?
   1. For the following statements related to organizational structure, tell me how strongly you agree or disagree with each. (strongly agree, agree, neither agree nor disagree, disagree, strongly disagree)
      1. *My organization has enough experience with mobile markets to implement the Veggie Van model*
      2. *My organization has enough staff to implement the Veggie Van model*
      3. *My organization has enough experience with taste testing/cooking demos to implement the Veggie Van model*
      4. *My organization has enough experience with nutrition education to implement the Veggie Van model*
      5. *My organization has enough Level of experience with data collection to implement the Veggie Van model*
      6. *My organization’s vision and mission align with the Veggie Van model SA*
      7. *My organization has enough support from Board of Directors/Advisory Board to implement the Veggie Van model*
      8. *My organization has enough support from supportive local government to implement the Veggie Van model*
      9. *My organization has enough funding to implement the Veggie Van model SA*
      10. *My organization has enough resources available, other than staff (e.g. office space, marketing materials), to implement the Veggie Van model*
      11. *Anything else? _______________________*
2. What kinds of infrastructure changes to your organization have been needed to assist with implementing the Veggie Van model?
3. *Interviewer to note answers from list below and probe on the following as time allows (may skip probes based on timing):*
   - 1. *Hire more staff – in process of hiring more staff*
     2. *Staff training – bundling/meal kit*
     3. *Staff training on taste testing/cooking demos*
     4. *Staff training on nutrition education*
     5. *Staff training on data collection*
     6. *Secure funding*
     7. *Secure resources, other than staff (e.g. office space, marketing materials)*
     8. *Anything else? _______________________*

**TENSION FOR CHANGE***

1. What might be some challenges or sources of tension within your organization in terms of implementing the Veggie Van model?
2. How has the Vegie Van program filled gaps in organizational practice/processes?

**GOALS & FEEDBACK**

1. Have you/your organization set goals related to the implementation of the Veggie Van model?
   1. How has your organization monitored/assessed progress towards implementation of the Veggie Van and related goals?
2. Have you/your organization set goals related to the implementation of a mobile market or other program, at the planning sites?
3. If yes, what are the goals?

**ORGANIZATIONAL INCENTIVES AND REWARDS**

1. What is your motivation for wanting to help ensure implementation of the Veggie Van model is successful?
2. Are there incentives or recognition from your organization?

**READINESS FOR IMPLEMENTATION: LEADERSHIP ENGAGEMENT**

*Ask questions to anyone other than the executive director of an organization (confirm organizational role as needed)*

1. How has leadership within your organization endorsed or supported the implementation of the Veggie Van model?
2. What level of endorsement or support have you seen or heard?
3. How has leadership at your organization been involved with implementing the Veggie Van model so far? If so, to what extent?
4. Yes, they know about our intentions to implement the Veggie Van model, but are not involved in the implementation process
5. Yes, they know about our intentions to implement the Veggie Van model and are involved in the implementation process
6. No, they do not know about our intentions to implement the Veggie Van model and are not involved in the implementation process
7. *Anything else? _______________________*
8. What kind of support or actions can you expect from leaders in your organization to help make implementation successful?
9. *Interviewer to note answers from list below and probe on the following as time allows (may skip probes based on timing):*
   - 1. *Financial (e.g. grant funds)*
     2. *Staffing - administrative*
     3. *Staffing – logistics/operations*
     4. *Strategic planning and problem solving*
     5. *Marketing and outreach resources*
     6. *Procurement and sourcing resources*
     7. *Navigating regulations and policy*
     8. *Anything else? _______________________*

**READINESS FOR IMPLEMENTATION: ACCESS TO KNOWLEDGE AND INFORMATION***

1. Now I’m going to list some Veggie Van trainings and/or materials that you may have participated in or received. Please provide feedback on each of the resources you have had access to:
   1. Research 101 [UB-hosted webinar]
   2. Veggie Van Toolkit Training [UB-hosted webinar]
   3. Data collection training [UB hosts prior to market launch]
   4. Farmers Register webinars [Perigee/UB-hosted webinar]
   5. Mobile Market Summit 2019
   6. Mobile Market Summit 2020 (as applicable)
   7. COVID calls/open calls
   8. In-service on Veggie Van Study provided by your organization to your staff

**CHARACTERISTICS OF INDIVIDUALS**

## **SELF-EFFICACY**

1. On a scale of 1 to 10, with 1 being not confident at all and 10 being very confident, please rate how confident you are that you will be able to successfully implement the Veggie Van model? (Probe: What made you choose this number?)
2. On a scale of 1 to 10, with 1 being not confident at all and 10 being very confident, how confident do you think your colleagues in your organization feel about implementing the Veggie Van model? (Probe: What made you choose this number?)

**OTHER PERSONAL ATTRIBUTES**[If completed interview at baseline, skip to “Process section”]***

The next set of questions is focused on you so we can gain a better understanding of what type of teams are best for running mobile markets.

1. How many years have you been working at *[organization]*?
2. What is your current position?
3. Administration
4. Market manager
5. Market staff
6. Evaluation team
7. Other: ____________________
8. What is the highest level of education you have completed?
9. Eighth grade or less
10. Some high school
11. High school graduate or GED
12. Trade or beauty school graduate
13. Some college
14. College graduate
15. More than undergraduate (some post graduate, post graduate, or professional degree)
16. What is your educational/professional background?
17. Before working with the mobile market, did you have any training or education related to your program?
18. *Interviewer to probe on the following after respondent has answered unprompted:*
19. *Public Health*
20. *Nutrition*
21. *Business management*
22. *Cooking*
23. *Agriculture*
24. *Marketing or Communications*
25. *Community Engagement*
26. How do you see yourself in relation to the communities served by your mobile market? *[Note to interviewer: do not read answers, but may confirm which they fall into]*
27. *I consider myself a member of the target communities (select one or more of the below answers)*
    - 1. *Lives in the same neighborhood*
      2. *Similar economic background*
      3. *Same race or ethnicity*
28. *I am not a member of the target communities, but I have been working with them for a long time and am trusted by community members*
29. *I do not consider myself a member of the target community*

**GRIT SCALE *[Skip this section if interviewee completed a baseline interview; only complete for new staff since baseline]***

1. Now I’m going to ask you to tell me a bit about your personality. Please feel free to tell me to skip any questions you don’t feel comfortable answering. For the following personality traits, please tell me if you feel the statement is: very much like me, mostly like me, somewhat like me, not much like me, or not like me at all.
2. New ideas and projects sometimes distract me from previous ones
   - 1. *Very much like me*
     2. *Mostly like me*
     3. *Somewhat like me*
     4. *Not much like me*
     5. *Not like me at all*
3. Setbacks don’t discourage me
   - 1. *Very much like me*
     2. *Mostly like me*
     3. *Somewhat like me*
     4. *Not much like me*
     5. *Not like me at all*
4. I have been obsessed with a certain idea or project for a short time but later lost interest
   - 1. *Very much like me*
     2. *Mostly like me*
     3. *Somewhat like me*
     4. *Not much like me*
     5. *Not like me at all*
5. I am a hard worker
6. *Very much like me*
7. *Mostly like me*
8. *Somewhat like me*
9. *Not much like me*
10. *Not like me at all*
11. I often set a goal but later choose to pursue a different one
12. *Very much like me*
13. *Mostly like me*
14. *Somewhat like me*
15. *Not much like me*
16. *Not like me at all*
17. I have difficulty maintaining my focus on projects that take more than a few months to complete
18. *Very much like me*
19. *Mostly like me*
20. *Somewhat like me*
21. *Not much like me*
22. *Not like me at all*
23. I finish whatever I begin
24. *Very much like me*
25. *Mostly like me*
26. *Somewhat like me*
27. *Not much like me*
28. *Not like me at all*
29. I am diligent
30. *Very much like me*
31. *Mostly like me*
32. *Somewhat like me*
33. *Not much like me*
34. *Not like me at all*

**PROCESS**

**PLANNING**

1. Can you describe the plan for implementing the Veggie Van model?
   1. Who was involved in the planning process?
2. Can you describe the plan for implementing a mobile market or other program, at the planning sites?

**ENGAGING: OPINION LEADERS**

1. Who are the key influential individuals to get on board with the implementation of the Veggie Van model? How have they been involved?

**ENGAGING: FORMALLY APPOINTED INTERNAL IMPLEMENTATION LEADERS**

1. Who leads the implementation of the Veggie Van model? How have they been involved?

**ENGAGING: EXTERNAL CHANGE AGENTS [HOST SITES]/ KEY STAKEHOLDERS***

1. On a scale of 1 to 10, with 1 being not supportive at all and 10 being extremely supportive, how supportive of your work are the community/host sites you are working with for the Veggie Van study (both market and planning sites)? (Probe: What made you choose this number?)
   1. In what ways are the sites you are working with helping your organization run a mobile market adopting the Veggie Van model?

**ENGAGING: CHAMPIONS [OTHER THAN HOST SITES]**

1. Other than your contacts at the community/host sites, are there other individuals outside your organization who you think will serve as champions in support of your mobile market? How have they been involved?
   1. In what ways have these individuals helping your organization run a mobile market adopting the Veggie Van model?

**ENGAGING: MOBILE MARKET CUSTOMERS***

1. What is your communication strategy for getting the word out about your mobile market in the community, more broadly?
   1. *Interviewer to probe on the following after respondent has answered unprompted:*
      1. *Online dissemination (e.g. social media, emails)*
      2. *Paper marketing (e.g. mailers, brochures, flyers) – print flyers*
      3. *Community meetings and presentations*
      4. *Press coverage (e.g. news, radio)*
      5. *Word of mouth*
2. How do you or your colleagues communicate with current and potential customers, including those that filled out interest forms, about the new/future mobile market?
3. *Interviewer to probe on the following after respondent has answered unprompted:*
4. *Online dissemination (e.g. social media, emails)*
5. *Paper marketing (e.g. mailers, brochures, flyers)*
6. *Community meetings and presentations*
7. *Press coverage (e.g. news, radio)*
8. *Word of mouth*

**EXECUTING**

1. Has the Veggie Van model been implemented according to plan?
   1. Why or why not?

**Interview Exit**

***Exit A***

Again, we would like to thank you for your participation in this Veggie Van interview. We may contact you for additional information – would that be ok?

1. Yes
2. No

If you have any questions about the Veggie Van Study, please feel free to contact us by phone at (XXX) XXX-XXXX or via e-mail at XXXXXXXX. We will reach out in approximately 2 months to schedule a 3-month follow-up interview with you or another representative at your organization. Thank you and have a great day!

***Exit B (If a follow-up call is warranted)***

Again, we would like to thank you for your participation in this Veggie Van interview. We were unable to complete all of our interview questions during this phone call. Would you be willing to schedule an additional phone call to speak with us? *[Note to interviewer: Proceed to scheduling follow-up phone call if the participant is willing. If they are unwilling, conclude phone call]* If you have any questions about the Veggie Van Study, please feel free to contact us by phone at (XXX) XXX-XXXX or via e-mail at XXXXXXXXXXX. Thank you and have a great day!
