# Supplementary material for: Protocol for the process evaluation of a mobile produce market intervention to increase fruit and vegetable consumption in lower-income communities: the Veggie Van Study
Source: Front Public Health. 2026 Apr 15;14:1760383. doi: 10.3389/fpubh.2026.1760383 (PMC13126556; doi:10.3389/fpubh.2026.1760383)
Supplement: Supplementary file 3 [file Supplementary_File_3.pdf]

Planning and Market Sites - Process Measures Form

Please complete the survey below.

This form helps the Veggie Van study team understand how the Veggie Van model is being implemented, what is working and what is not working, and which components best help business and customers' purchasing and dietary habits. When completing this form, think about the last calendar month (e.g., if it is currently the beginning of June, think about the month of May) that your mobile market visited a market site for the Veggie Van study.

Thank you for your time in taking this survey.

-

New questions may appear depending on your answers to certain questions.

Please be sure to answer every question completely, and do not click submit until all questions have been answered.

Please complete this survey for [month_1_arm_1][which_site]	
Is [month_1_arm_1][which_site] a market site or a planning site?	◯ Market<br>◯ Planning
Staff member completing form:	______________________
Current date and time:	______________________
For what month are you completing this survey?	◯ January<br>◯ February<br>◯ March<br>◯ April<br>◯ May<br>◯ June<br>◯ July<br>◯ August<br>◯ September<br>◯ October<br>◯ November<br>◯ December
How would you describe your mobile market model?	◯ Traditional in-person market set-up in the vicinity or within a host site<br>◯ Drive-thru pick-up at a host site<br>◯ Box pick-up model (similar to a community supported agriculture CSA)<br>◯ Home delivery<br>◯ Other (explain)
Please describe the "Other" MM model:	______________________

How many times was a mobile market program held at [month_1_arm_1][which_site] in the last calendar month? [This includes traditional in-person markets as well as adapted versions such as a drive-thru pick-up, home delivery, digital pre-order, or box pick-up models.]	◯ 0<br>◯ 1<br>◯ 2<br>◯ 3<br>◯ 4<br>◯ 5<br>◯ Other
Other number of times:	______________________________
Why was a market not held last month? [check all that apply]	☐ Planned seasonal break<br>☐ Inclement weather<br>☐ Vehicle issues<br>☐ Sourcing/produce issues<br>☐ Staffing issues<br>☐ Host site issues<br>☐ Other
"Other" reason that the market was not held:	______________________________
Additional comments:	______________________________
When the market was held last month, was it always located directly at [month_1_arm_1][which_site]? [For alternative mobile market models, such as delivery, think about if you consistently served the same community]	◯ Yes<br>◯ No, we had to adjust the location of this market site
Why did you have to adjust where the market was offered? [Check all that apply]	☐ Inclement weather<br>☐ Site-related issue [please specify]<br>☐ Needed to make the market more visible or accessible<br>☐ Other
Please explain the site related issues that you experienced.	______________________________
Please explain the "Other" reason you had to change the location of the market.	______________________________
How many market days did you have to adjust the location for this site?	◯ 1<br>◯ 2<br>◯ 3<br>◯ 4<br>◯ Other
Please explain the "Other" times of days you had to adjust the market location:	______________________________

Please briefly describe where this market site was moved to [This includes if you switched operations to a "virtual market," home delivery, or box pick-ups at your organization]:	__________________________
On average of the past month, how many paid staff members were present on market days at [month_1_arm_1][which_site]? (Do not include volunteers; for alternative mobile market models, such as delivery, think about the number of staff needed for operations)	◯ 0<br>◯ 1<br>◯ 2<br>◯ 3+<br>◯ Other
Explain "Other":	__________________________
On average, how many unpaid volunteers were present on market days at [month_1_arm_1][which_site]? (Include unpaid interns; for alternative mobile market models such as delivery, think about the number of staff needed for operations)	◯ 0<br>◯ 1<br>◯ 2<br>◯ 3+<br>◯ Other
Explain "Other":	__________________________
Where did you most often get produce for this [which_site] last month? You may choose up to 3 of the following options:	☐ Rescued/donated food (e.g., local retailer donates leftover produce close to expiration)<br>☐ Direct from farm (including your organization's own farms, if applicable)<br>☐ Farmers' market<br>☐ Leftover crops from farmers' fields (gleaning)<br>☐ Produce auction<br>☐ Wholesaler/distributor<br>☐ Other
Please explain the "Other" places you got produce from:	__________________________
On average, about what percentage of the produce offered at the market last month was locally sourced?	◯ 76-100%<br>◯ 51-75%<br>◯ 26-50%<br>◯ 25% or less<br>◯ None
Was a bundle offered at the market last month? [A bundle includes several produce items that are promoted and sold at a set/discounted price; the items do not necessarily need to be physically bundled together; they can include non-produce shelf-stable items; this includes meal kits and produce boxes]	◯ Yes<br>◯ No
How many days did the market offer a bundle last month?	◯ 1 time<br>◯ 2 times<br>◯ 3 times<br>◯ 4 times<br>◯ Other
Please explain the "Other" amount of days a bundle was offered:	__________________________

How would you describe the bundle that you offer?	◯ A pre-determined bundle of various whole produce items sold at a set price.<br>◯ A pre-determined bundle of whole produce items with shelf-stable cooking item(s) sold at a set price (e.g., a meal kit)<br>◯ Build your own bundle in which customers can choose a set number of whole produce items from several options for a set price. (e.g., "Choose two items from X, Y, and Z for $X"<br>◯ Other
"Other" way you would describe the bundle:	__________________________________
Do you incentivize the purchase of a bundle? (e.g., provide bonus items such as extra produce, non-local produce, dried goods, shelf stable cooking ingredients, or offering a discounted price)	◯ Yes<br>◯ No
Do you include a recipe with the bundle?	◯ Yes<br>◯ No

The next questions are about nutrition and cooking education for mobile market customers over the past month.

Was a food or nutrition lesson/activity (distributing recipes alone does not count as a lesson; informal and/or verbal education does count if it includes a learning activity) offered at [which_site] at least once last month? [This includes virtual/online nutrition education as long as it is promoted at this site; this does not include food demonstrations - we will ask about that later]	◯ Yes<br>◯ No
Was the food or nutrition lesson/activity conducted virtually, in-person (directly at the market), or a combination?	◯ Virtual<br>◯ In-person<br>◯ A combination of virtual and in-person
How often was a food or nutrition lesson/activity at the market at this site (not including food demonstrations)?	◯ 1 time<br>◯ 2 times<br>◯ 3 times<br>◯ 4 times<br>◯ 5+ times
Was a cooking demonstration or food sample conducted/offered at the market at [which_site] at least once last month? [This includes virtual/online cooking demonstrations as long as it is promoted at this site; (Check all that apply)]	☐ Food tastings (e.g., pieces of fruit or cut-up veggies)<br>☐ Cooking demonstration (with or without samples for customers)<br>☐ Recipe samples (with or without cooking demonstration)<br>☐ No cooking or food demonstration
Was the cooking demonstration conducted virtually, in-person (directly at the market), or a combination?	◯ Virtual<br>◯ In-person<br>◯ A combination of virtual and in-person

How often was a cooking demonstration or food sample conducted/offered at [which_site]?	◯ 1 time<br>◯ 2 times<br>◯ 3 times<br>◯ 4 times<br>◯ 5+ times
How often did you distribute a newsletter to customers last month?	◯ 1 time<br>◯ 2 times<br>◯ 3 times<br>◯ 4 times<br>◯ 5+ times<br>◯ We usually create a customer newsletter, but didn't have one last month<br>◯ We don't usually offer a customer newsletter
What percentage of customers do you estimate received a mobile market newsletter?	◯ All of the customers<br>◯ More than half of the customers, but not everyone<br>◯ Fewer than half of the customers<br>◯ Other
Please explain "Other" percentage of customers you estimate received a mobile market newsletter:	__________________________________
Were complementary services or events held at the same time as any of your markets last month? These may include events or services hosted by another organization, but in coordination with your market. Examples include the local health department tabling alongside the market doing blood pressure screenings, or distribution of Farmers Market Nutrition Checks, etc.	◯ Yes<br>◯ No
What type of complementary services or events was held at the same time as the market?	__________________________________
How often were complementary services or events held at the same time as the market?	◯ 1 time<br>◯ 2 times<br>◯ 3 times<br>◯ 4 times<br>◯ 5+ times
Has your organization or the host site initiated any new food or nutrition programming at [which_site]?	◯ Yes<br>◯ No
Please describe the new food or nutrition programming.	__________________________________
What type of pricing model are you offering at this market site?	◯ Free food distribution (produce is given away)<br>◯ Pay-what-you-can (no price is posted on items and customers pay based on means)<br>◯ Suggested price (suggested prices are posted but they are flexible based on customers' means)<br>◯ Sliding scale (prices are posted and determined based on customers' income or participation in benefit programs)<br>◯ Set-price market (prices are pre-set by your organization)<br>◯ Other

Please explain "Other" pricing model used:	______________________________
What type of pricing signage is placed at [which_site]? [check all that apply]	☐ Pricing (set or suggested price) signs placed in front of individual items<br>☐ A central pricing (set or suggested price) sign that lists all prices<br>☐ A central pricing sign that indicates sliding scale criteria<br>☐ Other [short answer text]<br>☐ No pricing signage is posted
Please explain "Other" pricing signage place at the site:	______________________________
How would you describe your prices at this market site?	◯ Below purchase price (we take a loss)<br>◯ At purchase price (we break-even)<br>◯ 1-10% markup from purchase price<br>◯ 11-20% markup from purchase price<br>◯ 21% + markup from purchase price<br>◯ Other
Please explain "Other" price description:	______________________________
Does this market site accept SNAP for payment?	◯ Yes<br>◯ No
Does this market site participate in any incentive programs to help reduce the cost of produce? Common examples include SNAP matching programs, Farmers' Market Nutrition checks, and Veggie Rx programs. However, this could include any incentives or discounts your organization has created or any regional or national incentive program you have chosen to participate in.	◯ Yes<br>◯ No
What type of incentives are accepted at this market site? This includes coupons, vouchers, free produce with purchase, non-food items; etc. [check all that apply]	☐ SNAP matching program<br>☐ Matching program of other assistance programs (Disability, Medicaid, Veterans)<br>☐ Farmers' Market Nutrition checks [Seniors or WIC]<br>☐ Veggie Rx or similar program<br>☐ Other
Please describe any "Other" incentives that are accepted:	______________________________
Has your market been using Farmers Register software?	◯ Yes<br>◯ No
About what percentage of new customers were registered in Farmers Register last month (e.g., name and phone number)?	◯ 76-100%<br>◯ 51-75%<br>◯ 26-50%<br>◯ 25% or less<br>◯ None

About what percentage of all customers that were registered in Farmers Register did you also collect demographic information for?	◯ 76-100%<br>◯ 51-75%<br>◯ 26-50%<br>◯ 25% or less<br>◯ None
What is the average number of customers that you estimate attended this market site each week?	◯ 0-25 customers<br>◯ 26-50 customers<br>◯ 51-100 customers<br>◯ 100+ customers
Please provide any additional comments	__________________________

This form helps the Veggie Van study team understand what community engagement and planning activities best predict a future viable market. When completing this form, think about the last calendar month (e.g. if it is currently the beginning of June, think about the month of May) your organization engaged with a planning site for the Veggie Van study.

Were planning activities or events held at [which_site]?	◯ Yes<br>◯ No
What type of planning activities or events? (Check all that apply)	☐ Focus groups<br>☐ Listening sessions<br>☐ Community advisory board meeting<br>☐ Other
Please explain the "Other" type of activity or event:	__________________________
How many planning activities or events were held in the past month?	◯ 1<br>◯ 2<br>◯ 3<br>◯ 4+
Has your organization or the host site initiated any new food or nutrition programming at [which_site]?	◯ Yes<br>◯ No
Please describe the new food or nutrition programming.	__________________________
Additional comments	__________________________
